# Why did he say that? Teaching physicians-in-training how to recognize hidden emotions in end-of-life prognosis conversations: an autoethnography

**DOI:** 10.12688/mep.19098.3

**Published:** 2022-08-25

**Authors:** John Stonestreet

**Affiliations:** 1 Hopecare, 740 4th St. N, Saint Petersburg, FL, 33701, USA

**Keywords:** end-of-life, prognosis, communication, breaking bad news, subconscious, emotions, self-awareness, ethics, truth-telling, shared decision making

## Abstract

**Background **: This article begins with two unconscionable end-of-life prognosis-related quotes from best-selling physician-author, Atul Gawande, and an unnamed doctor, asking: “Why did he say that?” The article then proceeds to answer this question by showing how physicians’ most common end-of-life communication blunders are rooted in their unexplored emotions. Healthcare’s only widespread communication training focused on examining the role of hidden emotions in influencing the flow of conversation is found in Spiritual Care’s “Verbatim” education modules. While the need for physicians’ emotional self-awareness for improved end-of-life communication has been identified in the literature, no one has explored how this need might be met by custom-tailoring Spiritual Care’s “Verbatim” education modules for physicians-in-training.

**Methods **: This article utilizes the qualitative research method of autoethnography to grant physicians access to the content and power of Spiritual Care’s “Verbatim” education modules for identifying hidden emotions in clinical communication.

**Results **: Using a profound personal example from the author’s firsthand experience of the suggested training tool, the “Verbatim” module is shown to grant revelatory self-knowledge and invaluable emotional intelligence. The same model then illuminates the physician cases.

**Conclusion **: Spiritual Care’s “Verbatim” education modules address universal issues of clinical communication and emotional self-awareness that are applicable to physician-patient/family conversations surrounding end-of-life decision-making. Customizing these communication modules for physicians-in-training may help to address physicians’ emotionally-triggered conversational miscues in end-of-life prognosis communication. Existing programs for complementary end-of-life communication training are noted, and it is claimed that a combination of each of these models, together with the proposed module, may be ideal. It is also admitted that no form of education or training can ensure ethical communication. Therefore the ultimate solution is to supplement communication training with real-time, third-party support and accountability. This can be achieved by the "Doctor Body Cam" intervention protocol, introduced here: https://aquila.usm.edu/ojhe/vol17/iss1/7/.

## Introduction

Dr. Atul Gawande is one of America’s favorite physician-authors, perhaps in part because he has the courage to confess when he puts his foot in his mouth, so that others can take note and learn from his mistakes. As reported in *The New Yorker* in 2010, when Sara Monopoli, an obviously dying patient, asked him for a frank reality check, “Am I going to die?”, Dr. Gawande replied with a bold-faced lie: “No, no. Of course not.” ^1^

Why did he say that?

He knew the truth that she was seeking, and he deliberately kept it from her. His dishonesty caused her to select harmful treatments that prevented her from maximizing family time in the weeks preceding her death. How could the same medical genius who (among countless other remarkable accomplishments) recently reconceptualized the entire field of public health en route to offering a disarmingly effective solution to COVID vaccination uptake, ^2^ be prone to such tragically regrettable and impactful communicative maleficence?

Before we proceed to answer that question, here’s one more example of a doctor communicating in a regrettable manner. Online healthcare consumer reviews are a suggested source for reporting of discrimination, ^3^ and I recently read the reviews for a critical care physician who is also trained in ethics and a member of a hospital ethics committee. Multiple families gave him one-star reviews (the worst possible rating option) for their conversations with him as their family members neared death in the intensive care unit (ICU). They reported that his response to their hopes and prayers for a miracle via divine intervention for their dying relatives was toxic to the extreme, leaving them feeling like he derided them as idiots for wasting their time with prayer in response to the news of a poor prognosis. One reported that after she endured his verbal assaults in the earlier prognosis conversation, he again stopped by the grieving family days later, just before the moment of death, venturing a mocking chuckle and bitterly cruel taunt, “better keep prayin’! Ha ha ha!” ^*^ The reviewer said that this was the “worst night of [her] life” and the doctor’s cruelty multiplied her suffering immeasurably.

Again, why did he say that? The fact that this doctor spends his days as a physician rather than a serial killer makes it clear that he wants to help people rather than torture them. So, once more: Why did he say that? (!)

For all of its amazing successes at masterminding disease processes, modern medicine has no reliable way of getting to the bottom of such unthinkable communication failures and understanding why they happened. Perhaps the only method of analyzing conversations that is capable of reverse-engineering such clinical communication faux pas is found in the Spiritual Care training program known as “Clinical Pastoral Education” or “CPE.” ^4^ CPE for Spiritual Care Providers is the standard, post-degree clinical training required for board certification and a career in Spiritual Care. Just as physicians embark on a clinical internship and residency after finishing their MD, Spiritual Care Providers complete a CPE internship and residency for approximately 15 months after completing their three-year Master of Divinity (MDiv) degree. While it may still be the best kept secret in healthcare education, there is a growing awareness of the value and adaptability of CPE, not only for physician/nurse-provided Spiritual Care, ^5^ but also more generally for leadership, emotional intelligence, and communication skills. ^6^ Having completed a CPE Internship and Residency at Duke University Hospital, and later dabbled in CPE Supervisory Training, I can speak about the program firsthand. In CPE, Spiritual Care Providers are trained in communication analysis for the purpose of conversation empowerment. Sitting in small groups led by a supervisor who is versed in a variety of behavioral theories, including my favorite, family systems theory, Spiritual Care Providers-in-training unveil and re-perform a verbatim account of a clinical interaction with a patient and/or family. In doing so, we expose ourselves to exploration and questioning by peers who have come to know us at some depth after hearing (through previous assignments) our family-of-origin narrative and learning something about the formative relationship dynamics that led to us becoming the person we are today. This dynamic peer review of our re-performed patient/family conversations allows others to ask of us the kind of question we just asked of Dr. Gawande and the unnamed doctor above: “Why did you say that?”

Invariably, conversational misfires can be traced to subconscious *emotional triggers*, commonly rooted in family-of-origin, or other formative, relationship dynamics. For example, a peer or supervisor might venture to the learner in the hot seat, “when you responded that way to this elderly woman patient, it reminded me of something you shared about your relationship with your mom. Would you like to explore that?”

Yes, this is deep stuff. And not everyone responds well to this kind of emotional rigor; it can be rather grueling. But it is designed to soften some of our unconscious hard edges and open us up to a more authentic and clear experience of ourselves and others. It can be thought of as a calibration of our self-monitored conversational “body cams” if you will, enabling us to grow in the direction of being able to self-regulate by acknowledging and bracketing emotional triggers in real-time so that our own subconscious doesn’t get in the way of holding space for the patient/family’s whole story, as difficult as it might be to hear.

When I speak of the patient’s story being difficult to hear, one example of this might be a patient grappling more deeply than she ever had with her own mortality after hearing from the doctor that her disease was terminal. Making room for the patient to explore her mortality might remind the listener of his/her own mortality, or perhaps his/her aging mother’s mortality, and s/he might unconsciously change the topic of conversation to avoid enduring these painful thoughts, saying something like this:

Well there’s nothing much we can do about our mortality; we will all die at some point, but all any of us really have is today; so what do you think is most important for you at this present moment?

Of course the sad irony here is that the patient was just trying to explore what was most important for her at this present moment, but she was tragically disenfranchised of this important exploration; so she obediently responds with something like this:

Well my son has always wanted the Bucs to win the Super Bowl, and I’m just excited for him that they finally have a chance to play for it this year. So I guess maybe that’s what is most important for me at this moment….

And the conversation then turns to her son’s love of football starting at a young age playing Little League. In a world without the hindsight provided by rigorous communication analysis, such as CPE’s “Verbatim” practice, these subtly disenfranchising conversational reroutes happen all the time without anyone ever realizing it.

Medicine tends to leave the soft skills of conversational refinement to the chaplains and let the physicians just focus on the science, but Dr. Gawande’s patient (quoted above) didn’t ask the chaplain, “Am I going to die?”, because she knew the chaplain didn’t have the medical knowledge necessary to answer the question. Similarly, it was the unnamed physician above, not the chaplain, who was tasked with delivering the grim prognosis to the families of dying patients in the ICU and fielding their responses about faith, prayer, and miracles. This is why I am particularly interested in marshaling CPE’s “Verbatim” approach to communication training for physicians who, by virtue of their role, bear the weighty responsibility of discussing matters of life and death (and related high-stakes healthcare decisions!) with very sick patients and their families.

We want to think of physicians as impervious to emotion in their cognitive functioning as objective medical scientists. But it has been said that the opposite is true. In the words of Dr. Danielle Ofri: “Understanding the positive and negative influence of emotions in the doctor-patient interaction is a crucial element in maximizing the quality of medical care.” ^7^ Harvard physician-scholar, Jerome Groupman, goes so far as to say: “Cognition and emotion are inseparable.” And: “The two mix in every encounter with every patient.” ^8^ Then, what mix of cognition and emotion, or, to put it another way, what hidden baggage might physicians be bringing to a prognosis and treatment conversation with the family of a likely dying patient? And what role might pedagogical innovation play in sparking insight that ultimately helps physician-trainees to be more fully present and truly open to a wider range of possibilities in these pivotal and sacred moments?

In addition to the question, “Why did he say that?”, we also seek to answer the equally important and related question: “Why did s/he *not* say that?” Prognostic misdirection or conflict assumes some level of engagement with reality, but the larger issue for prognostic communication may be physicians’ avoidance of reality altogether. Most cancer patients, for example, desire information about their prognosis, but physicians routinely skirt around the issue and withhold prognosis information, skipping ahead to treatment information as a forgone conclusion. ^9, 10^ This is a frighteningly enormous problem. One analysis of 590 advanced cancer patients found that out of the 71% of patients who wanted to know their prognosis, only 17.6% said that they were told. ^9, 11^ Patients tend to assume that the focus on treatment in lieu of prognosis information means that the chemotherapy being offered can actually cure their cancer, but this is very often not the case at all. ^12, 13^ Dubbing this tragic miscommunication as a holdover from the “dark ages of our field,” Harvard oncologist, Dr. Al-Samkari, sounded the alarm that this “misunderstanding is not the exception; *it is the rule*” (emphasis added). ^12^ After being assured by a senior hematologist/oncologist that this prevalent prognostic deception was not happening on purpose, but that most of these doctors actually believe that their patients can beat the odds, Al-Samkari asks which is worse, for doctors to lie to their patients or to lie to themselves? As we will see, this suggestion that doctors are lying to themselves again invokes the role of emotions underlying physicians’ prognostic communication. Although Al-Samkari and Patel ^14^ acknowledge the potential value of prognostic communication tools suggested by Abernethy *et al.*, ^9^ they agree with Loh *et al.* ^15^ on the greater importance of attacking the underlying issue: helping physicians overcome the emotional inertia causing them to avoid prognosis conversations altogether. Loh *et al.* ^15^ stress the importance of emotional self-awareness training for physicians, and Al-Samkari and Patel ^14^ advocate working with the attending physicians leading fellowship programs so that they can learn emotional self-awareness for themselves and then inculcate it in their fellows. But what should that emotional work with fellowship programs look like?

This complex issue of emotional self-awareness is quite resistant to simplistic solutions, which is why these pervasive problems continue to exist despite the triumphant forward march of medicine’s more technical successes. I have a medical education solution to propose; but to help you to understand the significance and efficacy of my proposal, I first need to take you with me on a personal journey, to give you a concrete, and hopefully moving, example of the transformative rigor of my proposed solution, before I apply it to the physician communication problems at hand.

My ultimate purpose for this article is to harness the power of Spiritual Care’s CPE “Verbatim” practice to propose a medical education module that could have prevented Dr. Gawande from telling that bold-faced lie he confessed above and perhaps also have prevented the unnamed physician’s more strident vocational discordance. But before we adapt CPE’s “Verbatim” practice for physicians-in-training, I will first demonstrate the tremendous power of this education module through a personal example from my own experience of it. As such, my case sets the stage for exploring (and continues to inform) the physician cases. By extrapolating from the insight gained through my personal experience of emotion-centric communication-analysis training in the context of CPE “Verbatims,” I seek to challenge medical educators to explore how novel modules of deliberate self-reflection for patient-facing medical students, residents, and fellows can help to better form “physician healers” ^16^ and transform the giving of healthcare. Drawing on relevant research interpreted through the lens of my own story, I ask medical educators to consider how unexplored emotions can unconsciously hamper physicians’ functioning in clinical determinations and related conversations with patients and families.

## Methodology

### Study design

While there is a growing awareness of the importance of emotional formation for medical students and tailor-made training to instill it, ^16^ there is no consensus on how this is accomplished, and concrete approaches to emotional intelligence training for budding physicians are few and far between. Therefore an autoethnographic ^17^ case study design was adopted. Previously used to address other efforts in education change, ^18^ autoethnography “describes an array of qualitative approaches centered on interpretive and/or critical methods.” ^17^ Most fundamentally, autoethnographers “write selves in relation to others.” ^17^ As such, autoethnography “seeks to describe and systematically analyze personal experience,” ^19^ and apply that insight to the analysis of other phenomena. Some who are unfamiliar with autoethnography as a *postmodern* research methodology want to put it in a familiar box and package it like any other *modern*, social scientific research method, with data collection, participants and sampling, data analysis, reproducibility and the like. But, “as a method, autoethnography is both process and product.” ^19^ Taking seriously the epistemological implications of postmodern contextuality, autoethnography prizes subjective knowledge as a reliable way of knowing and a vital complement to modern scientific objectivity. This does not mean that autoethnography is divorced from science and objectivity, just that it reappropriates these categories through a postmodern lens. Hence, autoethnographers “believe research can be rigorous, theoretical, and analytical *and* emotional, therapeutic, and inclusive of personal and social phenomena.” ^19^ Accordingly, in the philosophical tradition of Aleksi Losev’s *Dialectics of Myth*, ^20^ “[a]utoethnography, as method, attempts to disrupt the binary of science and art.” ^16^

Put in terms of a case study, authoethnography holds that the better I understand my own case, the more insight I might bring to understanding your case and other cases with similar threads. Consequently, this article first explores the author’s experiences of Spiritual Care’s “Verbatim” education module for emotion-centric sle of conversations. The author’s case study demonstrates the solvency of the proposed education module, and the insight gleaned from the author’s case is then applied to cases of problematic physician communication found in the literature. This case-by-case application demonstrates how Spiritual Care’s “Verbatim” education module can be customized for physicians-in-training.

### Selection of case studies

I selected my own case study not only because it illustrates the inner workings of CPE “Verbatim” practice, but also because, as you will see, some of the emotional dynamics revealed in my case are relevant to my analysis of Dr. Atul Gawande’s case. With that in mind, I take the liberty of using some descriptive subsections in my presentation of the cases to readers in order to highlight the conceptual issues that ultimately culminate in the application of my case analysis to Gawande’s case analysis. My case was narrated from previous “Verbatim” documents that I completed in the context of CPE Supervisory Training. ^†^ Given this article’s focus on physician training for *end-of-life* communication, the two physician cases were selected as paradigms of end-of-life decision contexts that are prone to physician communication mishaps. Because this article is particularly interested in the role of hidden emotions, both cases illustrate common emotional dynamics in end-of-life conversations. In one case Dr. Gawande has an emotional block that renders him unable to be honest with his patient about the reality of her impending death. In the other case, the unnamed doctor has an emotional block that renders him unable to empathize with families who are reluctant to give up hope for miraculous healing via divine intervention in the face of a terminal prognosis.

Dr. Atul Gawande’s case was found in an article he wrote in the New Yorker in 2010, ^1^ and the unnamed ICU doctor’s case was found in public online reviews of his professional medical practice. Snippets from other cases in the medical literature are interspersed to inform the analysis of the two main physician cases.

## Case studies

### My Case: “Unearthing Hidden Shame”

In a previous life, I was training to become a “CPE Supervisor.” As introduced above, CPE is a form of clinical training that seeks to inform and improve the quality of care communicated by Spiritual Care Providers in their conversations with patients and families through an action-reflection-action model of learning. As a clinician, you are asked to reflect on a particular conversation you have had with a patient and/or family in the hospital, through a written verbatim account of “he said,” “she said,” “I said.” Included in the “Verbatim” write-up, you are also asked to analyze the conversation through a variety of theoretical lenses. Ultimately, you re-perform the conversation for a small group of peers, led by a CPE Supervisor. And you seek input from your peers and the supervisor to inform your efforts at self-reflection.

During one such “Verbatim” report, I was recounting my conversation with a patient who had within one week’s time gone from the best day in her life, her wedding, to the worst day in her life, her hospitalization. Suffering a life-threatening health event, she barely managed to drag herself across the floor to phone for help and escape the clutches of death, only to become exasperated by a nightmarish experience of customer service on her first day in the hospital. In reflecting on my verbatim account of this conversation, I was asked by my CPE Supervisor to put myself in the place of the patient and wonder for a moment if I had ever experienced a similar turn of events where things quickly unraveled from the highest of highs, where I felt on top of the world, to the lowest of lows, where my world was falling apart. My ego was a bit bruised by the question because I thought I had already demonstrated in my verbatim account how well I had empathized with the patient, so I didn’t understand why I was being asked to recount such an experience, as if I hadn’t yet demonstrated empathy for her and needed to undergo a remedial thought exercise to open myself to the possibility of having solidarity with the patient’s experience. But, I did my best to place my wounded ego aside and accept the invitation for a vulnerable self-reflection. The outcome surprised me.

I found myself recounting an experience that began one Friday night in September, 2004. I was attending seminary outside of New York City at the time, and I had just received the news that a summer of working 100+ hour work weeks had culminated in me winning a scholarship contest that would help defray the cost of tuition. I met up with a couple of seminary classmates to hit some bars in NYC and celebrate. After we boarded the metro, I joyously handed each of my buddies a $100 bill to kick-start them into the celebratory mood that I was already feeling. I then called my sister, ^††^ who was living in the city at the time, to let her know that we were headed in her direction for a night out, and may end up crashing at her apartment if we missed the last train back to Westchester. My sister’s reply caught me by surprise and immediately jolted me out of my celebratory mood. She said something to the extent of, “If you come here, I will not be coming out of my room, and if I never see you again, there is a note on my computer. Goodbye.” Click, dial tone. The only sense that I could make out of my sister’s words was that she may be contemplating suicide. She had been managing life with a serious eating disorder for many years, and I was one of the people she leaned on most for support, so I was accustomed to accompanying her through difficult times, but it seemed that on this night she had hit a new bottom.

After repeatedly phoning to reach her housemates and my parents and getting no answers, I picked up the phone again and this time called 911 to ask for advice on how to respond to a loved one who has made a statement that sounds potentially suicidal. The 911 operator ignored my request and instead kept asking me for an address. I tried to explain that I wasn’t calling to request an ambulance but rather a referral to a resource who might provide some guidance through a conversation over the phone. The operator said that she wasn’t allowed to provide a referral until she first registered the call by inputting the address of the person for whom I had concern. I naively complied. As soon as I divulged my sister’s address, in hopes of being transferred to a suicide counselor for advice, the operator simply said: “an ambulance is on the way to the premises.” I tried to explain that I was not asking for an ambulance yet, just for some initial advice, but the operator said, “sorry, that’s what we do,” and repeated, “an ambulance is on the way to the premises.” At this point, my goal then became to try to beat the ambulance to my sister’s apartment, but the metro had not yet arrived at Grand Central, and I was still an additional cab ride away.

When I finally reached my sister’s place, the ambulance had already arrived and she was responding to their interrogation in a rage, telling them they were crazy and demanding that they leave her home. At that point I wondered, “what if they do leave and she has already taken a bottle of pills and there is no way to know and nothing I can do?” So I spoke up with a feeling of desperation and said: “she said there was a note on her computer.” One of the EMTs opened her laptop, and there was a Word document saved to the desktop entitled, “If I die.” It was a note of goodbye to her family and friends. That was all the paramedics needed to spring into action. They then forced her out of her apartment, against her will, and into the ambulance to be taken to the hospital for an examination. As they dragged her out the door kicking and screaming, with me following close behind, she looked back at me intently and said like a dagger, “if I survive this, I will kill you, and then I will kill myself!” The emergency room visit proved anticlimactic as we did a lot of waiting, followed by a quick physical once-over to determine that her life was not in danger, and we eventually hailed a cab back to her apartment. It turned out that her concern about dying was not the result of having taken a bottle of lethal pills for the purpose of suicide, like I feared from her words and her note. Rather, she was speaking in response to intense physical pain from her most recent, rapidly spiraling bout of bulimia, and she simply feared that she had unintentionally pushed her ravaged body over the edge. Upon experiencing this unprecedented level of physical pain, she feared she may be actively dying. So the note, “If I die,” was written in case it was too late for her to recover from her latest bout of bulimia and her body was perhaps shutting down for good.

When we arrived back at her apartment, and I lay down next to her in her bed that night, there were still those awful words hanging in the air in the hallway just outside her room: “if I survive this, I will kill you and then I will kill myself!” While I knew she had spoken under great duress, a part of me was still afraid to close my eyes because I kept hearing those words, and I feared for us both. Sleep did come. The next day we took a slow walk together in the city and stopped for lunch. All I remember from the conversation was that in the very moment that I started to feel comfortable, as if we had moved through a difficult terrain together and were arriving safely at the other side, she looked up with the same intensity of her threat the night before, except only in a lower volume, the hush of which was more chilling than the previous scream: “What you did to me last night was the worst thing anyone has ever done to me in my whole life; it was worse than being raped.” For emphasis, she again repeated: “what you did to me was worse than when I was raped.” I was shell-shocked. All I had done, in my mind, was to try to keep her alive when she had spoken of dying, to try to protect her as best I could.

All of this I recounted to my CPE group, chuckling with a smile on my face. I looked up at the supervisor who had invited me to reflect and said, still smiling, “so, yeah, I guess I have had my own experience that started out as a celebration and ended up a little different at the end of the day.” The supervisor responded that when I have had a chance to process that experience in therapy I might be able to narrate it without the emotional disconnect of smiling and that working with this story could prove to be very powerful for my pastoral identity and my clinical functioning. I was then asked if I had ever discussed the experience with my sister and shared how painful it had been for me. I replied that I maintained a close relationship with my sister but that I did not feel the liberty to confront her in that way. Soon after that comment, the training session was over, but my supervisor’s suggestion of confronting my sister lingered on in my mind.

In my next two Verbatim presentations over the weeks that ensued, the emotion that kept surfacing for me was *shame*. In my conversation with two daughters grieving after having just been asked to consider whether they wanted to change their dying mother’s code status from “full code” to “do not resuscitate” in the event that her heart stopped, I found myself turning inward. Although I wanted so badly to be there for these grieving women, and while, in many ways I was still there for them, a part of me had turned away from them and turned inward in shame. Here is how it happened: We had been talking together for a few moments when I asked,

“What sustains you and keeps you going at times like this?”

One daughter responded, “We’re not super religious, but we do have some belief.”

The other daughter joined in, “Family is what really helps us cope.”

“Are there any other members of the family, do you have any other siblings?” I asked.

“No, Dad is gone, and our only brother just died,” said one. “So it’s just Mom and us and our kids,” said the other.

Suddenly, seemingly out of nowhere, I felt overwhelmed by shame. At that moment, I wanted to kick myself, duck my head between my legs and disappear. And why? Well, as irrational as it sounds in hindsight, I immediately blamed myself for directing the conversation in such a way that felt to me at the time like I had added insult to injury for these sad, grieving sisters. Here they were already grappling with their mom’s impending death, and my contribution to the conversation functions to shed an even darker light than was already present. My internal dialogue went something like this:

Not only have they just been told that their Mom’s heart may stop at any time, and it may be pointless or even harmful to try to bring her back, but now I’ve made a bad situation even worse by reminding them that their best resource for coping, family, is in awfully short supply as their father is long dead and their brother also *just died*. So my role in the conversation was to shine a big light on how their best coping resource, their dear family, is vanishing from sight right before their eyes, one after another until they find themselves all alone with no source of hope. The stupid chaplain opened his mouth and only made it worse.

Looking back now, I can see now how irrational my thought process was in that moment. It wasn’t my fault that my inquiry revealed more sad news. That news was no news to them, and sharing it with me did not make their situation any worse. If anything, it only helped them to be able to offload and process with me yet another layer of what they were already dealing with. Why then all of the shame? Why was I so deathly afraid that I would make a bad situation even worse? The following week, I presented another Verbatim, and I uncovered more shame and more irrational fear that I might make a bad situation worse. One of the CPE Supervisors then asked me: “Was there ever a time in your past when you tried to help someone in a bad situation, and you felt like you made it even worse?” No answer was needed. Everyone in the room already knew the weight I carried from being told by my sister that my efforts to save her life were experienced by her as more torturesome than rape.

Fast forward to a few months later, I had the opportunity to act on my supervisor’s challenge to consider confronting my sister about this experience as a part of my efforts to seek healing for myself. My sister was visiting from Italy, where she now lives and works leading idyllic, private wine-country tours while also providing online personal coaching for girls with eating disorders in the US. I shared with my sister how the event had surfaced in the context of my training.

“Jo,” I said, “Could we talk about something that’s been bothering me?”

“Sure,” she said, “What’s up?”

“Jo, I wanted to tell you that something I have been realizing in processing my conversations with patients and families through this training program is that I keep struggling with shame.”

Beginning to cry, I continued:

When I find myself trying to care for people who find themselves in a really difficult situation, I keep feeling scared and ashamed that in trying to help I am somehow going to make it worse for them. My supervisor asked me if I’ve ever made a situation worse when I was trying to help someone, and what came to my mind was that day in the city when you told me that when I called 911 when I thought you were going to die, that what I did to you was worse than when you were raped. (Crying harder now) I just wanted to help you Jo, and I was so scared—I didn’t know what to do. I wasn’t trying to inflict anything bad on you; I didn’t have any kind of agenda to harm you. I didn’t want to be at the hospital that night any more than you did. I wanted to be out with my friends celebrating. But you told me if I never saw you again there would be a note on your computer and I thought you were dying, Jo, and I didn’t know what to do. I just did the best I could, and you told me it was worse than rape.

Joanna reached out and put her arms around me like a warm blanket of love, and looking deeply into my eyes, she said:

“John I am so sorry I said those things to you, and that you have had to carry that pain…”

“Thanks, Jo,” I said, continuing to cry. “I’m so afraid that I’ll make it worse for patients and families just like I made it worse for you.”

“John, I was very sick, and I was just lashing out. *You didn’t make it worse*.”

At that point the floodgates really opened and I cried like a little baby while my big sister held me and comforted me. Then there was one more thing I needed to say:

“Jo, you told me that night that if you lived through the ambulance experience, you were going to kill me and then kill yourself.”

“John, I’m so sorry,” she said with great care.

I continued through the tears: “I was afraid to go to sleep that night because I feared you were going to *kill me*, Jo.”

“Oh John, please forgive me, I am so sorry,” she said, holding me tighter.

“I do forgive you, Jo. Thank you so much for hearing that and caring for me.”

As my crying settled down, my sister loosened the grip of her hug, pulled back to where she could look me in the eyes, and said:

I want you to know that when I talk to people about that weekend, I remember it very differently than you do. What I remember is your unconditional love and care. I don’t talk about my brother who made it worse; I talk about my brother who made it better because he stuck with me through my hell and wouldn’t leave me to suffer alone.

Hearing this redeeming assurance from my sister reminded me of something a CPE Supervisor had said that really stuck with me. Looking back on my verbatim sessions surrounding shame, this one thing that was said to me stands out the most. As I sought feedback from the regional CPE Supervisory Training group in grappling with the conversational paralysis triggered by my shame in overwhelming clinical encounters, this Supervisor turned to me and said, with authority and care, “just because you’re helpless, doesn’t mean you’re worthless.” Those may have been the eight most memorable words from my first unit of CPE Supervisory Training: “ *Just because you’re helpless, doesn’t mean you’re worthless*.”

Hearing from my sister twelve and a half years later that my desperate efforts to care as best I could in a helpless situation were meaningful rather than harmful helped to ingrain in me a new internal dialogue which opened the space for new reflexes to replace the paralysis of misplaced shame. That new internal dialogue can be summed up by the motto: “ *Just because you’re helpless, doesn’t mean you’re worthless*.” I benefited a great deal from this revelation, and I have also witnessed a number of peers from diverse backgrounds growing in their own self-knowledge and emotional intelligence through CPE. Teaching this motto through a standard classroom lecture would be nothing like hearing it in the CPE-Verbatim-induced moment of intense, contextual self-reflection. Trying to learn such a lesson through a lecture course alone might be like having a blindfolded nurse playing Pin-the-Tail-on-the-Donkey with a Band-Aid and firmly attaching it to my nose in response to a cut on the back of my leg. The solution is offered, but it doesn’t exactly connect with the problem.

I believe that my story illustrates the difference between education, training, and *formation* and that this distinction could be critical for the future of medical education. Education gives you information about a given topic; training teaches you how to do something; formation functions at a different level by addressing the personhood that you bring to your professional duties. Formation avails you of the self-knowledge necessary for personal growth. By putting you in touch with who you are in relationship to what you do, formation avails you of the opportunity to evolve toward the person and the professional who you feel called to be.

Revisiting and reflecting deeply on your own words and feelings from previous conversations provides an invaluable gateway into the journey of personal and professional formation. Perhaps it is no accident that three of the world’s leading physician communication trainers suggest writing down everything you can remember from a challenging clinical conversation, emotions included, and analyzing it in hindsight, ^21^ although they also provide the qualification that emotional self-knowledge is beyond the scope of their communication training practice. ^21^ This is where the assistance of a CPE Supervisor comes in. If the CPE Verbatim analysis can be said to help the learner give birth to emotional self-knowledge, then the CPE Supervisor cultivates a small group environment in which both s/he and the peer learners function as midwives to the birth-giving of emotional formation for the learner on the hot seat.

Since my first experience with CPE more than 13 years ago, I have found myself wondering if CPE “Verbatims” may well be the best kept secret in medical education. I would love to see other clinicians benefit from similar experiences of emotional formation. Of course, each clinician has a different emotional makeup and a unique personal and professional journey, so emotional “growing edges” will vary widely, person to person. With that acknowledged, I am also curious: What are some common clinical contexts in which similar types of emotions might tend to surface for similar types of clinicians? In the forthcoming exploration of the physician cases, we will attempt to answer that question in the process of imagining what a “Verbatim” analysis might have revealed about each case.

### Outward-Directed Emotions: The Unnamed ICU Doctor Case

In my previous doctoral research on physician responses to families hoping for a miracle via divine intervention in response to poor prognoses, I found that a physician’s level of frustration with purportedly irrational family members can have an influence on the physician’s strategy for navigating the conversation with family. In the extreme example of the unnamed doctor quoted above, his bitter sarcasm seems to indicate that he may have been more than just frustrated at the miracle-hoping families, perhaps irritated or even angry. These strike me as contextually outsized emotions, disproportionate to anything one might rationally expect from his passing encounters with families in the intensive care unit (ICU). I imagine that if this physician had the opportunity to write out or ad lib a verbatim account of his conversations with the families he mocked for praying, and discuss his words and actions with a supportive peer group, he may come to the realization that his extreme emotions of irritation and/or anger were actually coming from somewhere other than his brief encounters with these vulnerable grieving families he barely knew.

Perhaps there is an arrogant, condescending, and/or pushy “holy-roller” in this physician’s family-of-origin, a “Bible-thumper” kind of character, that sibling who weaponizes faith to gain power in relationships rather than using it to grow in humility, kindness, and unconditional love. If this doctor was experiencing transference from that formative relationship and/or projecting something from that relationship onto these random families in the ICU, that could make some sense out of his otherwise unexplainably aberrant behavior. This is the kind of insight clinicians can gain from performing a “Verbatim” of clinical encounters in a supervised peer environment, matching words with related emotions, and engaging in a little self-exploration to discover where the emotions are really coming from. With this self-knowledge in hand, the critical care physician in question could easily come to a realization that he needed to research and intentionally implement a considered communication protocol ^22^ for families hoping for a miracle via divine intervention, to replace his default susceptibility to knee-jerk, emotionally triggered outbursts in these contexts.

### Inward Emotions and How to Identify Them

While some physicians surely struggle with intense outward-directed emotions like frustration, irritation, and/or anger with patients and/or families like this one did (whether for good reason or out of emotionally triggered projection/transference), I am more interested in emotions like shame that are directed inward, toward oneself. I have found that these kinds of emotions can be harder to identify and therefore have a greater potential to fly under the radar and go undetected, or at least unexplored for a longer period of time. But, is this hunch rooted in reality? Could doctors’ self-directed emotions, like shame, have an impact on their medical determinations or at least on how they share medical information and navigate conversations surrounding treatment decisions with patients and families?

As previewed above, according to Dr. Danielle Ofri, attending physician at Bellevue Hospital and Clinical Professor of Medicine at New York University School of Medicine, the answer is “yes.” Despite popular images of stoic objectivity, doctors have emotions like everyone else, and doctors’ emotions impact both their medical care and their conversations with patients and families making pivotal health care decisions. In her book, *What Doctors Feel*, Ofri contends that while doctors’ hidden inner feelings might be less widely examined than their thoughts, they may be “at least as important.” ^7^ Citing Dr. Jerome Groupman’s book, *How Doctors Think*, Ofri quote’s Groupman’s observation that “[m]ost medical errors are mistakes in thinking,” and “part of what causes these cognitive errors is our inner feelings, feelings we do not readily admit to and often don’t even recognize.” ^7, 8^ The subject of medical errors is beyond the scope of this article, which is more interested in clinical communication than technical performance. But, returning to the above question about hidden baggage, if doctors’ emotions function as hidden baggage so powerful that it causes physical missteps as concrete as technical medical errors, then it may be safe to assume that doctors’ feelings would have at least as strong an impact on complex treatment decisions and related conversations with patients and families. Indeed, Ofri contends that the hidden “emotional layers in medicine.... can often be the *dominant players in medical decision-making*, handily overshadowing evidence-based medicine, clinical algorithms, quality-control measures, even medical experience” (italics mine). ^7^ Confirming our conjecture on hidden baggage, Ofri stresses that emotions can dominate medical decisions “without anyone’s conscious awareness.” ^7^

If this is so then it raises a number of ethical issues such as prognostic truth-telling, related patient access to health information, shared decision-making, and informed consent, especially at or near the end of life. “[E]nd-of-life decisions,” says Ofri, “can be strongly influenced by a doctor’s emotional state.” ^7^ Drawing on neuroscientist Antonio Damasio’s description of emotions as the “unstoppable humming” or the “continuous musical line of our minds,” and dubbing this emotional music the “basso continuo,” Ofri seeks to understand how this “continuous bass line” of physicians’ emotions impacts the overall production of healthcare as experienced by the patient.

For the patient-provider communication aspect of healthcare delivery, a CPE “Verbatim,” like the one I reflected on above, creates space for this kind of analysis. In a typical Verbatim format, the “he said,” “she said,” “I said,” account of words articulated in the conversation under analysis goes in the left-hand column of the Verbatim template, while the accompanying emotions felt in connection with those words are detailed in the adjoining column on the right. This combined account of the words and accompanying emotions of the conversation creates a full-fledged song, if you will, complete with emotional score, allowing for a considered reflection on how the words and the “continuous bass line” of emotions combine to create a particular performed piece, the conversation under analysis. It was in that second column where I identified the emotion of shame in my conversation with the two sisters whose mother was dying, shame so irrational in any objective analysis of the conversational flow but so powerful and paralyzing for me at that moment. I can still remember assigning the emotional “score” to the words of my Verbatim. I sat staring at the words in the left-hand column for some time, taking myself back to that moment in my mind, standing there in the ICU, listening for the emotional music, and allowing the feelings to finally speak their way onto the right-hand column of the Verbatim template. This is the kind of deliberate self-reflection I am advocating for physicians-in-training.

### The Need for an Education Solution and Barriers to Implementation

There is a demonstrated need for what I am suggesting. Drs. Ofri and Groupman are not alone in their insistence on the importance of emotion for physicians navigating complex medical decisions with dying patients and families. The critical need for emotional self-awareness training to bring oncologist prognostication out of the “dark ages” ^12^ was identified above by Al-Samkari and Patel ^14^ and Loh *et al.* ^15^ Additionally, in a study of physician residents’ interactions with surrogate decision makers, Reckrey, *et al.* ^23^ found that “[r]esidents have complex and emotionally significant interactions” and “experienced significant emotional burden.” As a result of their findings, Dr. Reckrey and colleagues recommended that “educational efforts seek to help residents *understand their own emotions* and the ethical beliefs *that underlie the roles they adopt*” in their interactions with healthcare decision makers (italics mine). This is a need that CPE “Verbatims” can fulfill.

While it cannot be said that no medical student or physician has ever written and analyzed a verbatim account of a clinical conversation in a small group context (likely, the “Physician-Self: Reflection in Practice” aspect of Dr. Rita Charon’s vision for “Narrative Medicine” had something akin to this kind of formation in mind) it may be safe to say that this is far from a consistent, widespread, and properly guided/mediated practice in medical training. ^24^ Over the years, I have learned through the grapevine that a handful of innovative CPE Supervisors have offered CPE for non-chaplain clinicians to great success (such as the CPE program for intensivists at MassGeneral Hospital for Children ^5^ and the successful CPE for healthcare providers pilot study at Yale-New Haven Hospital), ^25^ but these programs have been very few and far between. Physicians are primarily trained to understand and manipulate body parts, fluids and functions. But, it is the physicians’ conversations with patients and families that have the greatest influence on healthcare decisions and even entire treatment trajectories that can sometimes frame those individual decisions.

One of the obstacles to physician participation in CPE as it is currently practiced is that CPE students, being Spiritual Care Providers, clergy or divinity students for the most part, are required to articulate their own theology at the outset and explore through “Verbatims” how it operates as a guiding framework for their clinical practice. Some in the medical profession may either not have any particular theology (if they are not particularly religious or not disposed to faith at all). And even if they are people of faith, they might not be well versed in the theology of their faith community or simply might not feel comfortable articulating their theology in writing and verbally to their CPE group. I wonder if this gap might be filled by personal spirituality and/or the medical humanities. In place of one’s personal theology as the chosen form of theoretical starting point or organizing principle for one’s approach to patient/family communication, the subset of medical students and physicians who do not have or wish to articulate a personal theology might choose to speak in broader terms about their approach to spirituality or, rather, might choose a text from the medical humanities as a symbol for the ideals they seek to bring to the practice of medicine. Using literature, for example, one medical student might say, “I want to be like the physician in this particular novel,” or operating from the negative, “I want to be the exact opposite of the physicians in these particular short stories.” Using philosophy, one might say, “I want to be a physician who completely escapes Foucault’s critique. I want to unmask forces of power and create pathways of freedom for patients and families in a way that would make Foucault proud.”

Having 1) shown how CPE verbatims unearth hidden emotions, 2) established the importance of emotion and communication for physicians, 3) articulated the need for CPE-like “Verbatims” for medical training, and 4) explored the example raised by the unnamed ICU doctor’s unkind quote above, we will now 5) work our way back to the opening quote of this article and apply everything we have learned to answering our question of Dr. Gawande above: “Why did he say that?” As already discussed, this question stands in parallel to the related question of why oncologists (among other doctors) so commonly avoid prognosis conversations with patients and families: “Why did s/he *not* say that?”

### Hidden Emotions in End-of-Life Prognosis Talk: The Case of Dr. Gawande

Duberstein *et al.* ^26^ suggested that “[c]onceptual models of DIALs [discretionary interventions at the end of life] might benefit from more explicit statements about *the role of emotions* (emphasis added).” If we can understand why Gawande lied to his patient about her impending death, this key may unlock the mystery of why so many curative-focused doctors struggle so mightily to open up about terminal prognosis possibility or likelihood, earlier in the treatment trajectory, when patients have more time to get a fuller benefit out of hospice. How might emotional formation through communication analysis be useful for physicians who, like Gawande, struggle to acknowledge the elephant in the room with their dying patients and families?

If Dr. Gawande was to write a verbatim account of his conversation with Sara Monopoli, with the words of the conversation in the left-hand column and the emotions he was feeling at the time in the right hand column, what emotions might Dr. Gawande have been feeling when Sara asked him, “Am I going to die?” and he responded with, “No, no, of course not.”? Drawing from my experience and research, the emotions that come to mind when I think about physicians communicating with dying patients and families facing difficult end-of-life treatment decisions are fear/anxiety, helplessness/worthlessness/shame, hope, and ambition. The underlying/hidden emotions here are fear/anxiety and helplessness/worthlessness/shame. Regarding fear/anxiety, we witnessed in the introduction to this article how an inexperienced chaplain might redirect the conversation with the patient away from her exploration of her own mortality, and then the two go on to talk about football instead. We observed that this was likely the chaplain’s own subconscious fear or “death anxiety” ^26^ doing the talking, either his/her fear/anxiety regarding their own mortality or his/her fear/anxiety regarding, for example, their aging mother's mortality, given the demographic overlap with the elderly woman patient. Like the chaplain example above, Dr. Gawande could have also redirected his patient, Sara’s, question about dying away from the truth of her situation because of his own anxiety/fear of death for himself and/or others in his family. Fear of death and death anxiety have been identified as potential triggers for burdensome discretionary interventions at the end of life ^26^, like the futile and harmful experimental chemo suffered by patient Sara as a result of Dr. Gawande's dishonesty. So it could have been fear and/or death anxiety that short-circuited Dr. Gawande’s conversation with Sara Monopoli. But those are not the only considerations. What about shame?

How might some physicians struggle with shame, and how might their shame interact with their own hopes and ambitions and with the hopes and ambitions of patients and families that they shape and respond to?

In her chapter titled, “Burning with Shame,” Dr. Ofri quotes psychiatrist and former dean of the University of Massachusetts School of Medicine, Dr. Aaron Lazare, describing shame as “an emotional reaction to the experience of failing to live up to one’s image of oneself.” ^7^ This is exactly what happened to me in my personal story above. Going back to my narrative of shame rooted in my long-assumed failure to help my sister that night in NY City, I will share that my image of myself that served as a backdrop for that story was one of helper, and ultimately saviour/hero within the relationship dynamics in my family-of-origin. From an early age, I managed conflicts between the other members of my family, absorbing into my body the angst between my siblings and my parents. In my previous CPE units, I worked on other aspects of this outsized and delusional self-image and how it evolved over my continued education and formation. Suffice to say here that a part of my shame from that incident with my sister was rooted in my failure to live up to an unrealistic and unhealthy image that I had of myself and my role within my family-of-origin, where I put too much pressure on myself to be a saviour/hero for others. How might my experience be relevant for a physician with a similar emotional makeup?

Dr. Ofri’s treatment of physicians’ shame is almost exclusively focused on shame resulting from technical medical errors, and the related failure to live up to one’s image of oneself as a competent doctor. She wonders “why is it that doctors feel their entire sense of self at risk when they admit errors”? Ofri’s answer is the “culture of perfection in medicine that fosters a strictly binary analysis: either you are an excellent doctor or you are a failure.” ^7^ While I do not disagree with this analysis, it seems to me to have an overly extrinsic focus. In addition to the extrinsic factor of medical culture, what intrinsic factors might cause doctors to “feel their entire sense of self at risk when they admit errors” such that “either you are an excellent doctor or you are a failure”? I wonder if there are any (or perhaps many?) doctors who, like me, have developed a self-image that is influenced by the way they functioned in the relationship dynamics of their family-of-origin. How might a savior/hero complex, compounded by the culture of perfection in medicine, impact a physician’s internal battle with shame? And how can that shame influence their interactions with patients and families surrounding complex treatment decisions? Cracking that code and unraveling that thread may have a tremendous impact on the future of doctor-patient/family communication surrounding trajectory-altering medical decisions where patients’ and families’ experience of dying hangs in the balance.

As detailed above, from my own experience speaking with dying patients and their families, there were times when I was unable to separate the helplessness of the situation from the assumed worthlessness of my personhood, presence, and care. I wonder how many doctors sometimes feel helpless to effect an adequate cure and, like me, subconsciously translate that feeling of helplessness to a feeling of worthlessness. Suggesting that doctors might be uniquely prone to shame, Dr. Ofri opines that “[b]ecause shame is so global and its consequences so devastating, human beings automatically erect walls to hide their shame, making it one of the most challenging emotions to examine, much less confront.” ^7^ If shame is so difficult to examine and confront, I wonder how many doctors don’t even consciously realize that when a patient’s condition starts to slip beyond their medical reach, they begin to battle shame, feeling helpless and therefore worthless. After all, what use is there for a healer who can’t heal you?

Tipping us off to another possibility for the hidden dynamics constituting Gawande’s inner struggle, one very insightful physician identified the “healer as hero” complex operating within herself and came to grips with how this false self-image impacted the way she approached end-of-life decisions with patients and families. In her book, *Extreme Measures: Finding a Better Path to the End of Life*, Dr. Jessica Zitter confesses her emotional need to “be a hero” and “rescue patients from the brink of death.” ^27^ What about all the inevitable moments when Dr. Zitter and others like her are unable to rescue a dying patient? If shame is defined as a failure to live up to one’s self-image, and Dr. Zitter wanted to be “a hero” how can shame not sometimes eat away at doctors who fit Zitter’s emotional profile? And how could that battle with shame, hope, and ambition become hidden baggage that a doctor carries into future patient/family encounters? As Dr. Zitter confesses, this is an especially important question to consider at the end of life when patients and families are clinging to hope as the road ahead darkens. If physicians might be particularly prone to shame as the healer/hero self-image is threatened by losing a battle against death, how might a physician’s unexamined emotion impact the journey of hope for dying patients and their families? If the doctor is ashamed to confess that despite his/her best efforts, death may likely prevail, then patients and families won’t have the information they need to recalibrate their hopes or at least consider planning for the worst as they continue to hope for the best.

Indeed, consistent with the results of an ethnographic study tracing this same dynamic, ^28^ Dr. Zitter reveals that when the time comes for that difficult conversation, all parties—doctor, patient, and family alike—often avoid the obvious. Instead of addressing the elephant in the room, everyone pivots toward discussions of more questionable treatments that can often increase suffering and complicate the dying process. Here is a short personal narrative revealing exactly how Dr. Zitter first came to terms with this dynamic in her own practice:

When I was a young attending [physician] I had been asked to put a large catheter in someone’s neck. She was dying. And I went into lifesaving mode. Right before we were getting ready, I looked up and I saw this nurse in the doorway, and she looked at me locking eyes with me and said:
“Call the police. They’re torturing a patient in the ICU.”
My heart dropped into my stomach, and I realized, “Oh, my gosh, she’s right. What I’m doing right now is not gonna help her. It’s not gonna get rid of this disease that’s killing her. And I don’t wanna do that anymore.” ^29^

This courageous nurse single-handedly put Dr. Zitter in touch with her emotion-based treatment decisions, acting according to her confessed hero-complex and using hope and ambition to try to live up to that delusional self-image while protecting herself from the inevitable feelings of worthlessness and shame that she later understood can accompany situations of helplessness for someone with her emotional makeup. By consciously acknowledging her shame, and struggling against it to embrace the solidarity of shared helplessness with patients and families, ^30^ Dr. Zitter has since discovered what “shame resilience” theorist, Brene Brown, describes as the welcome emotions that are the opposite of shame: “empathy, connection, power, and freedom.” ^30– 32^

What about Dr. Gawande? How might shame, hope, and ambition have played into Dr. Gawande’s failure to give an honest answer to his dying patient’s question? As referenced in the introduction above, here is the broader context for the previously quoted words of NY Times best-selling author/surgeon, Atul Gawande, when he knew beyond a shadow of a doubt that a patient was nearing death:

I said that it had not been possible to remove all the disease [through surgery]. But I found myself almost immediately minimizing what I’d said.
“We’ll bring in an oncologist,” I hastened to add. “Chemotherapy can be very effective in these situations.”
She absorbed the news in silence, looking down at the blankets drawn over her mutinous body. Then she looked up at me.
‘Am I going to die?’
I flinched. ‘No, no,’ I said. ‘Of course not.’ ^1^

Reflecting on Gawande’s confession, it is pretty hard to argue with Loh *et al.*’s ^15^ contention, affirmed by Al-Samkari and Patel ^14^ and Campbell *et al.*, ^33^ that “most high-stakes treatment decisions are influenced by psychological processes outside of conscious awareness,” and that “self awareness can be better incorporated into clinical training,” through the “use of stimuli that tap into nonconscious processes.” This is what is accomplished in a “Verbatim” assignment where you are asked to write out a given conversation to the best of your memory, reflect on your words written in the left-hand column, and list your related emotions next to those words in the right-hand column. This practice might reveal for Dr. Gawande that such a regrettable misstatement was some combination of what was, in one sense, his boundless hope and ambition, but in another sense, and more accurately, his fabricated hope and ambition, and perhaps ultimately in truth, his feigned hope and ambition, all serving to protect himself from the shame of failing to live up to his false self-concept as the hero who should be able to help find a way to save the day in the end. It has been contended that doctors mislead patients regarding prognosis because of how deeply they care about their patients ^9^ or because they really do believe their patients will beat the odds, ^12^ but Al-Samkari’s observation ^12^ that they are either lying to their patients or lying to themselves shines light on the ultimate reality of their subconscious wrestling with underlying emotions like fear/anxiety, and/or hope/ambition, and/or helplessness/worthlessness/shame.

## Discussion

After a Verbatim education module helps a physician-in-training to come to grips with their underlying emotions surrounding difficult prognosis disclosure, then therapeutic interventions like Loh *et al.*’s suggestions ^15^ for self-talk may be appropriate and very helpful:

*I want to fix this, but I cannot. Yes, it hurts me, too. I can reframe failure as an opportunity to do good. I can tolerate the uncertainty of not knowing how long this person will live*.

And,

*I can disclose the prognosis without falling apart and worrying that others may hate me. I must be honest because it will lead to better patient outcomes. I can provide information that patients want and need but just enough to make informed decisions and not bludgeon them with the inevitable*. ^15^

Absent abnormally high self-awareness combined with a powerfully well-timed intervention, like Dr. Zitter’s nurse threatening to call the police, or a CPE Verbatim unveiling shame’s role in botching treatment decisions, these kinds of unconscious emotional reflexes can operate under the radar for an entire career’s worth of incomplete prognosis explanation and inadvisable treatments for droves of physicians, like surgeons and oncologists (among others). Since shame is such a buried and hidden emotion, doctors are known to blame patient/family unreadiness (which is admittedly real but can often be better negotiated) ^22, 34^ for the doctor’s tragic inability to overcome their own emotions and address the elephant in the room. Cauley *et al.* report that surgeons have performed full-on operations that “they knew would not benefit the patient to give the family time to come to terms with the patient’s demise.” ^35^ When I hear that jarring admission, alongside Dr. Zitter’s similar confession, like Dr. Gawande, that sometimes for doctors, “offering [patients] more [treatment, even if it may do more harm than good] feels like being a better advocate” and serves as evidence “that they are really *trying*,” ^27^ I can imagine how the patient’s and/or family’s avoidance of reality presents a whole other dynamic for the doctor to manage alongside his or her own emotions. But I also wonder if that is sometimes, at least in some small part, the hidden baggage of unexamined physician emotions doing the talking. As one who has benefited from CPE Verbatims and hopes to share those benefits with physicians-in-training, I want to be able to one day tell a doctor who is tempted to offer a medical procedure, treatment, or technology that could be counterproductive to the care of a dying person and family that there are other effective ways to show that you are “really *trying”* to provide the *best care possible*. As demonstrated so poignantly by palliative care physician/evangelist, Ira Byock, in his book entitled, *The Best Care Possible*, ^36^ sometimes just sitting with a dying patient and/or family together, even if only for a brief moment, united by the solidarity and community of shared helplessness, or making a joint visit together with a caring chaplain, ^30^ might be the best way to show that you really care.

Remember: *Just because you’re helpless doesn’t mean you’re worthless*.

Four remaining points before concluding with Limitations and Future Research:

1) The feeling of helplessness can also be a factor in doctor-patient/family conflicts in the ICU when the doctor suggests “compassionate extubation,” known colloquially as “pulling the plug,” and the family is resistant since they are still holding out for a miracle via divine intervention. Here, the emotional tables are turned from the Gawande example above, and these doctors achieve their psychological self-concept by “saving the day” in a very different way than curing. With the goal of a peaceful death for the dying patient, these doctors work to dial families back from commonly burdensome end-of-life heroics. The above quote of the unnamed doctor was an extreme example to illustrate outward expressions of strong emotions. More commonly in these conflicts, doctors are not so much angry and sarcastic as frustrated, or irritated, and/or guardedly judgemental ^22, 30^. Rather than mocking the family like the unnamed doctor above, they will often take a more constructive approach and try to persuasively reason with families who are hoping for a miracle via divine intervention ^22, 29^, or simply avoid them altogether ^22, 37^ as they are deemed “irrational.” ^22, 38^ But the inability of these doctors to discover within themselves the desire to combine prognostic transparency with compassionately joining in solidarity with these families’ unlikely hopes has been dubbed an “intellectual ruse for emotional avoidance.” ^22^ In other words, the reflex against providing a nuanced, point-of-care, third-party prayer for the family’s hoped-for miracle, and communicatively embracing the strong divergence between hope and expectation, can be yet another way of (like Drs. Zitter and Gawande) avoiding the feeling of helplessness, ^22^ rather than embracing it, ^30, 39^ and discovering in the embrace of helplessness an emotional release of “empathy, connection, power, and freedom.” ^31, 32^ For many physicians, this is at first a counterintuitive element to person-centered communication surrounding "breaking bad news," but it can be ingrained and become second nature through training ^22, 40^.

2) VitalTalk ^41^ and Ariadne Labs ^42^ are already offering simulation-based, and other forms of electronic medical record-based, train-the-trainer, physician communication trainings, providing for practicing physicians (and probably for medical trainees) something at least somewhat akin to the CPE Verbatim modules I suggest, especially in that they target end-of-life communication; only they tend to be more skills-based and medical record format-based, focusing more on the “what?” than on the “why?” I think there is a place for both methods, with the others perhaps being more workable as continuing medical education (CME) for established physicians and the more self-reflective CPE "Verbatim" modules. Perhaps more effective for the emotional formation of physicians-in-training. Another underutilized, transformative method for providing in-depth, impactful communication training for medical students is to invite real-live patients in for open, frank dialogue with students in the classroom. ^43^ I believe this method would be symbiotic and synergistic with CPE Verbatim modules. And it would be especially helpful earlier on in medical school when students are not yet seeing patients. It may serve to set the table for a richer CPE Verbatim experience as students progress to patient-facing training.

3) Admittedly, widespread marshaling of CPE Verbatim education modules for physicians-in-training is an ambitious suggestion for the future of medical education. But even if this plan succeeds, there will be a variation in the extent to which each physician is open to engaging it to maximum benefit. Like the other existing end-of-life communication training programs, it will inevitably fall short of preventing all of the tragic instances of communication maleficence like the ones we witnessed in the opening quotes. Duberstein *et al*. ^26^ showed that physician comfort with medical paternalism is independently associated with chemotherapy use at the end of life. No form of training can be fully relied upon to make this magically go away. Relatedly, these authors also reference a supply-side “technological imperative” in late-stage medicine that is equivalent to the military “technological imperative” seen in total warfare, where no measure can be spared in the face of impending death. This kind of knee-jerk treatment imperative (elsewhere referred to as the “scientistic imperative”) ^34^ may be insurmountable without the imposition of external communication accountability. Such accountability was attempted for hematology/oncology fellows by Al-Samkari. ^12^ As a member of the Hematology and Medical Oncology Milestones 2.0 Committee of the Accreditation Council for Graduate Medical Education (ACGME), “which seeks to update and define the competencies necessary for a hematology-oncology fellow to graduate,” Dr. Al-Samkari “proposed adding the assessment of regular, honest, and full disclosure of prognosis and treatments to patients to the communication milestones.” Initially, the entire committee unanimously agreed on the measure, but it was later revoked as quickly as it was added. The reason?

Too many attending oncologists do not do this themselves, everyone concluded.
How would program directors evaluate whether fellows were doing this when the attendings themselves do not do it reliably? It would be like *the blind leading the blind* (emphasis added). ^12^

Comparable to the pre-Institutional Review Board (IRB) milieu for research ethics, the field of clinical communication ethics cannot be expected to spontaneously self-regulate. Therefore, I would contend that the only real answer for reliable ethical solvency for high-stakes physician-patient/family conversations is to devote the requisite communication training to third-party communication specialists. These third-party specialists can then be inserted into doctor-patient/family conversations, whether virtually or in-person, to ensure and to notarize shared understanding between doctors, patients, and families. ^34^ Think about Cauley *et al.*’s admission above that surgeons are actually cutting into the flesh of dying patients for no other reason than to buy time for families to come to grips with a terminal prognosis. ^34^ How can this confession be interpreted as anything other than a desperate plea for third-party communication support? These third-party communication specialists will provide for clinical communication the same level of ethical accountability that IRBs provided for research. This “Doctor Body Cam” intervention protocol is detailed in a recent article intuitively linked from DoctorBodyCam.com. ^34^ In that piece, I reconsider Dr. Gawande’s fateful lie to a dying Sara Monopoli and her husband Rich, and I detail how a third-party communication specialist could have easily prevented Gawande’s tragic debacle. Like the IRB did for research ethics, the “Doctor Body Cam” protocol is shown to solve for the future of end-of-life (and other high-stakes) communication ethics. But, the better that physicians are trained in communication, the better they will cooperate with third-party support, and the more seamless and effective the conversations will be for patients and families. For that reason, I would like to see a combination of communication training via patient/family classroom visits early in medical school, CPE Verbatim modules for patient-facing medical students, residents, and fellows, and VitalTalk and Ariadne Labs training for practicing physicians, as well as the linchpin, “Doctor Body Cams” for physicians-in-practice. ^34^ This combination would best combat the status quo likelihood that unexamined physician emotions can negatively impact the doctor-patient/family relationship and/or the determination of regrettable and unnecessarily onerous end-of-life treatment decisions and trajectories.

4) Speaking of body cams and the racial justice issues they invoke, CPE Verbatims may also provide a uniquely effective form of Implicit Bias education for physicians-in-training. There is a growing awareness that racial and ethnic health disparities may be less attributable to biological “nature” and more prone to medical “nurture,” or lack thereof, than previously thought. ^44^ Perhaps requiring peer and supervisor analysis of separate and comparable verbatim reports from conversations with patients and families of all races might help physicians-in-training to get better insight into their own unconscious biases. To borrow a line from the old children’s song, “Jesus Loves Me,” CPE Verbatim modules may help to unveil the extent to which it might truly be said of any particular physician: “Red and Yellow, Black and White; they are precious in his[/her] sight. Yes, [the doctor] loves me.”

## Limitations and Future Research

Now, I will address the limitations of my analysis/interpretations and corresponding suggestions for future research. Perhaps the most obvious limitation of what I have put forth here is that it is largely hypothetical in nature. I would like to contend that, however hypothetical it may be, not only is my analysis accurate, but it can even function as an interpretive key that unlocks the mysteries behind the two most common and impactful emotionally-driven communication failures affecting the quality and cost of end-of-life healthcare. And if that is not enough, I also claim to have developed novel interventions that solve both of these paradigmatic problems. ^22, 34^ Given such a wide gap between the hypothetical nature of my analysis and my bold claims for its significance, the need for implementation research is clear.

But what kinds of implementation research might I suggest? Notice that in my above contention for the significance of this article, I only referred to it as an “interpretive key.” Notice also that my "novel interventions" citations do not include a citation for "Verbatim" education modules. Indeed the primary intervention I would suggest for a randomized clinical trial (RCT) in response to the hidden shame blocking physicians’ terminal prognosis disclosures (that was revealed in this article) is not the “Verbatim” education exercise detailed here but the third-party “Doctor Body Cam” protocol ^34^ referenced above.

While I would love nothing more than to engage in the sacred task of interacting personally with medical students/residents/fellows who are open to testing out the “Verbatim” module and reporting back on their experience (which itself is an important form of research), I do not see this exercise as the best approach to large scale solvency for the emotionally-driven physician communication failures discussed in this article. This is due to the second limitation to my analysis in this article which is closely tied to my point three above that “there will be a variation in the extent to which each physician is open to engaging [the ‘Verbatim’ exercise] to maximum benefit.”

While it may seem like a subtle distinction, I want to take a step further and add that in addition to physicians’ *openness* to engaging the “Verbatim” exercise to maximum benefit, there is also the more fundamental limitation of physicians’ *capacity* for doing so. Let me explain. In my hypothetical analysis in the cases above, I assume an “aha” moment where the doctor “gets it,” and perhaps even more importantly, I also imply an assumption that this will translate into concrete changes in clinical practice, where the physician sees the detrimental emotion arising in real-time, side-steps and brackets that reflex, and then successfully self-initiates an effective conversational reroute. Could Dr. Gawande make that leap? Absolutely. His body of work clearly indicates that he already has. And the same goes for my reference to Dr. Zitter who confessed to the hero complex that previously drove her irrational interventions of harmful heroics. ^27^ But Dr. Gawande and Dr. Zitter are not your average physicians. They are thought leaders and exceptional superstars to say the least. In pointing that out, I am not making a dig at the intelligence of the average doctor. More fundamentally, I am commenting on the reality of what doctors are trained to do: fight disease. Imagine for a moment that doctors are wind-up toy cars. When you wind them up and set them down, what do they do? They fight disease.

Both this observation and the related question of "capacity" I have borrowed from two forbearers. Twentieth century medical ethicist, Paul Ramsey, in his classic text, *The Patient as Person*, ^45^ identifies the “question of questions” for end-of-life medical decision making within the doctor-patient/family relationship. Loosely paraphrased, the question is this: Can we reasonably expect to find within the same person, both the main vocation to fight disease and also the added vocation of taking the initiative with patients and families to introduce the question of when it is time to stop fighting disease and focus on putting affairs and relationships in order in preparation for the best possible experience of death? In addition to Ramsey’s “question of questions,” which I think raises the issue of physicians’ prognostic capacity, I borrow the provocative term, “capacity” itself, from another twentieth century medical ethicist, Dr. Jay Katz. In response to an early 1980s United States Presidential Commission calling for shared [medical] decision making (SDM) in the doctor-patient relationship, Dr. Katz names physicians’ “incapacity" for truth-telling due to most “doctors’ disregard of uncertainty.” ^46^ Here, it seems to me that Katz is provisionally answering Ramsey's "question of questions" in the negative. As a psychiatrist, Katz would not throw around the term “incapacity” lightly since that is something psychiatrists formally assess. Also trained as a lawyer, Katz understood the legal implications of the medical designation of incapacity or "incompetence," a term he also uses in calling his fellow physicians' prognostic and SDM capabilities into question. Katz knows that if patients who are declared "incompetent" for the task of medical decision-making are replaced by third-party "surrogate" decision-makers, then the same might necessarily follow for physicians, the majority of whom have an “incapacity” ^46^ for prognostic truth-telling (Regarding this prevalent incapacity, Dr. Nicholas Christakis wrote an entire book detailing his extensive research on the prevalence of physicians’ prognostic shortcomings ^47^). And Katz's legal chops would also make him inherently sensitive to the dangers of replacing physicians' role in sharing end-of-life decision-making with patients and families. Namely, there could be a legal danger of removing the task of medical decision-making from the doctor-patient/family relationship altogether and placing it into the hands of third-party bureaucracies who are external to the doctor-patient/family relationship. I think this is why Katz suggests that medicine resort to "limping" very slowly and everso cautiously in the direction of figuring out exactly how to comply with that 1980s presidential commission's vision for the future of medical decision-making.

Now nearly forty years later, it might be said that American medicine has not limped all that far, the growth of palliative care and hospice notwithstanding. This is why it again bears repeating that Dr. Al-Samkari finds his fellow physicians' prognostic disclosure capabilities still limping in the "dark ages." ^12^ When Al Samkari suggests that if doctors are not lying to their patients then they are lying to themselves, that assertion begs the question of capacity.

This is not to say that medical students, residents, and fellows have nothing at all to gain from "Verbatim" education modules, but that complete solvency to the problems raised in this article requires a multipronged approach. As stated above, the linchpin is the third-party “healthcare notary” who notarizes shared understanding between doctors, patients, and families for end-of-life and earlier difficult healthcare issues. ^34^

To summarize my vision for future research, I seek collaborators for the following three forms of research. First, I would like to deliver a Visiting Lecture on this article for medical students and Grand Rounds for practicing physicians. Afterwards, I would like to circulate a survey for feedback on the training method as well as the underlying conceptual issues. Secondly (and I envision that the first suggestion might most naturally lead to the second), I would like to pilot-test the Verbatim practice detailed above with patient-facing medical students, residents, and fellows, circulating a follow-up survey, especially soliciting open-ended responses. Third, I seek collaborators for a program of research that moves in the direction of major RCT's testing the quality and cost outcomes of third-party “healthcare notaries” ^34^ inserted into the doctor-patient/family relationship to ensure prognostic truth-telling and shared medical decision-making. My main role in the research collaboration would be to provide the training for the third-party “healthcare notaries” delivering the intervention.

Why am I confident that after nearly forty years of “limping” through the wilderness in search of the Promised Land of prognostic truth-telling and shared decision-making (SDM), that it is now time to take these ideas and run with them? Because the role of the “healthcare notary” ^34^ walks the tightrope that Dr. Katz suggested we “limp” toward mastering, without erring on either side of that fine line and falling into an abyss. Here's why: the healthcare notary addresses the issue of physicians' widespread emotional incapacity for prognostic truth-telling and SDM *without overcorrecting for that problem by removing the locus of the medical decision outside of the doctor-patient/family relationship*. This is because the notary is not imposing some external criteria for supposedly valid healthcare decisions but instead serving as a witness to the reality of a shared understanding of critical issues, between doctor, patient, and family. The healthcare notary's only role then is to act as a structural facilitator to the success of the all-important and irreplaceable doctor-patient/family relationship for prognosis and SDM. A specific example of what that might look like is found in the “Doctor Body Cam” protocol piece. ^34^

How does this vision fit into the current landscape for SDM research in the health services research community and the related and long-awaited physician uptake of SDM more broadly? For readers not heavily embedded in the SDM research community it bears mentioning that some of its leaders published an article ^48^ in 2020 predicting a “dystopian” future for SDM if it continues along its current trajectory. That trajectory lacks a viable pathway to physicians' widespread adoption of SDM, making many if not all of the SDM research community's marvelously constructed "decision aids" significantly underutilized.

This is because, in my opinion, the SDM research community looks almost exactly like the "research ethics" community would look like in a world without the Institutional Review Board (IRB). How can you get anyone to adopt your suggestions for ethical practice without a mandate? This is why the suggestions of that 1980s presidential commission on SDM have gone largely ignored for nearly 40 years: there was/is no mandate. For “research ethics,” the IRB was a mandate because you cannot conduct research without it. Requiring *notarized*, truthful prognosis disclosure and *notarized*, ethical SDM (incorporating the prognosis disclosure) before being legally permitted to initiate “informed consent” would be a mandate. Then suddenly all of the marvelous, context-specific "decision aids" created by the SDM research community would be grabbed up and used everywhere. And that for the same reason that researchers do whatever is necessary for IRB approval: it is mandated as a requirement for moving forward.

Here's to a utopian future for SDM with prognostic truth-telling as its first step.

## Data availability

### Underlying data

All data underlying the results are available as part of the article and no additional source data are required.

## Footnotes

^*^ This quote is paraphrased to protect the privacy of the doctor whose public review could otherwise be discovered through an online search of the direct quote. Suffice to say, the direct quote is no more flattering to the doctor than the paraphrasing.

^†^ a three+ year program certifying CPE Supervisors to deliver the previously-referenced hospital-based CPE internships and residencies

^††^ I have received permission from my sister to share this story.
