# Supplementary material for: Why did he say that? Teaching physicians-in-training how to recognize hidden emotions in end-of-life prognosis conversations: an autoethnography
Source: MedEdPublish (2016). 2022 Aug 25;12:32. Originally published 2022 May 4. [Version 3] doi: 10.12688/mep.19098.3 (PMC10828552; doi:10.12688/mep.19098.3)
Supplement: Peer review history

# Reviewer report: Reviewer response for version 3

**DOI:** 10.21956/mep.20680.r33652

**Published:** 2024-01-30

**Authors:** Anthony Herbert

**Affiliations:** 1 The University of Queensland, Saint Lucia, Queensland, Australia

This was certainly an interesting article to read and the idea of teaching through "Verbatim" analysis and reflection is very worthwhile.

 The article is quite long and could be made more concise.

 The manuscript adopts a "narrative approach" and the author speaks in first person.

 The themes of emotional awareness and how this impacts clinical communication and decision making is very worthwhile.

 Case studies from the author's personal life, Gawande and also an anonymous online feedback about an intensivist are used.  What is the ethics of using online feedback about a physician in a manuscript?

 The notion of a real-time 3rd party support and accountability tool in "doctor body cam" was also helpful within the introduction. This was referenced in another article. I didn't get a full appreciation of what this was in the current article.

 There were some opinion rather than fact in the paper e.g. the positive affirmations of Atul Gawande in the 2nd paragraph after the question "Whey did he say that?" was not necessary in my honest opinion.

 There is benefit in acknowledging the impact of family of origin, and other personal dynamics in how we communicate.

 The risk of distraction in moving away from emotionally laden topics was also highlighted positively.

 The role of emotions in how we communicate is a key theme of the manuscript. Perhaps this could be honed in a revision of the manuscript to make it more concise and focused?

 Evocative language is often used and could be curtailed somewhat .e.g. speaking of hidden agendas, rather than "baggage".

 The question "Why did he / she not say that?" is helpful to contemplate.

 It took me a little while to work out the author was a chaplain and not a physician. Albeit a chaplain with significant experience of healthcare. It could be helpful if this was made clearer earlier in the article.

 The author used a auto-ethnography method and describes this in the context of other literature. The "verbatim" methodology is also outlined. The post modern context also helps to frame this paradigm of research.

 As the author moves through their various verbose results and discussion section, the various case studies are informative. However, there could be some culling of evocative and excess wording.

 There were some helpful themes and ideas raised in the results section.

- The idea of hidden emotions and emotional block impacting clinical communication.
- How to respond to a family who are hoping for divine intervention in an end of life situation.
- The use of the question "What sustains you to keep going at a time like this?"

As the Results section develops, there are some helpful themes related to:

- How we can make things worse by trying to help.
- The role of the "internal" emotion of shame.
- "Just because you are helpless doesn't mean you are worthless".

"The pin the tail on the donkey" analogy was perhaps not helpful though and could be omitted.

 The role of small groups and peer support as part of the proposed "Verbatim" teaching methodology sounded helpful. Is there any literature that could inform this further?

 The differentiation between internal and external emotions was also helpful.

 I wondered if the author could differentiate between curing and healing as a paradigm that doctors could use to approach how to provide care when there is no longer curative options. It is still possible for a doctor to provide healing, even if the patient dies. I was surprised the author did not mention this. Just an observation.

 The notions of "heroism" and "perfectionism" in medicine, as potential barriers, to the provision of holistic and altruistic care was helpful. It did make me reflect on the hero-martyr-victim cycle that health professionals can engage in (that may lead to burn out) and no doubt has its routes in family of original, personality and also the health care education system.

 The idea of hope and ambition was also helpful to raise. I wondered if the author could tease out the implications of this more for both the patient, as well as the help professional.

 The Discussion starts with some helpful comments on "Self-talk". However, this seems out of place, as this is the first time self-talk has been introduced. Perhaps summarising the themes developed in the Results section could be more helpful here.

 The "body-cam" concept is a helpful one but perhaps could be explained more concisely for someone who has not heard the concept, or had the opportunity to read the referenced paper.

 Review of the work by Vitaltalk and also the Ariadne Labs (Serious Illness conversation) was helpful.

 In terms of other methodologies and limitations, I wonder if the author could comment on the research methodology of "conversation analysis" which video-records real-time clinical encounters.  Also, could the author comment on the role of ethnography as a research tool in areas of clinical literature?

 The Limitations and Future Research section focuses more on future research, and discusses implementation science. Perhaps the limitation of case studies could be mentioned here.

 The manuscript does have a summarising paragraph at the end. Perhaps this could be changed to a conclusion. My main take from reading the paper (which did take some time) was that in addition to skills and content, as well as assistance from electronic medical records in documenting conversations, there is also a need to be aware of emotions in communication. The Verbatim approach seems a novel way of doing this, and its use is demonstrated through 3 case studies. There is also linkage with the "Dr Body Cam" approach here.  

 The author discusses shared decision making in the latter stages of the manuscript and perhaps this could have been introduced earlier.

 Another example of the author's use of evocative language or hyperbole, would be in describing Gawande and Zitter as "superstars". I think the term thought leader is more appropriate.  The description of Gawande and Zitter as "superstars" is ironic given the author's thoughtful reflection on being a hero in healthcare.

 The author does give a suggestion of how their research could progress, including broader education and pilot interventions. It would seem both qualitative and quantitative feedback from participants would be an empiric place to start in terms of research. I also found it ironic, in terms of the author's observations of the short comings of evidence based medicine, that their conclusion was that a research program should ultimately end with a randomized controlled trial. Food for thought.

 I think the author raises some helpful concepts. There could be merits in reducing the word count so the key concepts are more apparent. And also to minimize the use of evocative language as I have outlined above (with examples).

 I didn't fully understand the notion of a "health care notary". Could the author's please explain further, or perhaps leave out, as it didn't seem central to the tenets of the paper for me. Is the health care notary to be used in clinical training, or real life clinical encounters. It almost seemed a separate paper in itself?

 I conclude the manuscript with 2 further questions for the author that they could consider in a revision of the manuscript.

 How do we manage the situation where emotionally intelligent doctors are more likely to self select by attending the proposed training.  Whereas, those who really need this training may not see its value. The author does partly answer this question in terms of introducing the training at medical school, and also trying to introduce a mandatory component (similar to health research ethics approval).

 How would the author respond to the following situation: A patient is at the end of life and the clinician feels there are no benefits in offering further intervention. They explain this to the patient and their family. Unlike in the examples given in the manuscript, the clinician is honest about the poor prognosis, and is open to not suggesting further non-beneficial treatment. In fact, they recommend palliative care. The family come back, and say they understand what the doctor is saying, but they feel there is still uncertainty in this situation. Both the patient and the patient's partner feel they had good quality of life before this health encounter, and would like to try one more time. How do you balance the patient's request for further treatment, with the clinician's preference to not provide treatment, in this context?

 Also, is it wrong for a family to ask for more time in intensive care, to give time to recover from a potentially reversible condition, to have time to process what is happening, or to say good byes and undertake spiritual rituals?

 Would it be wrong for a surgeon to offer an invasive treatment that would not be curative but could? ...give time to recover from the  condition for a time, to have time to process what is happening, or to say good byes and undertake spiritual rituals.

 Perhaps we are moving from communication to ethics with these questions. However, the emotional aspect (and potential bias) which is a central tenet of the author's paper does provide a link here. Could the author make a brief comment on the role of clinical ethics, and its interplay with clinical communication?

Is the case presented with sufficient detail to be useful for teaching or other practitioners?

Yes

Is the work clearly and accurately presented and does it cite the current literature?

Partly

If applicable, is the statistical analysis and its interpretation appropriate?

Not applicable

Is the case’s background and context in sufficient detail?

Yes

Are all the source data underlying the results available to ensure full reproducibility?

Yes

Are the conclusions drawn adequately supported by the results?

Yes

Reviewer Expertise:

Communicaton, Palliative Care

I confirm that I have read this submission and believe that I have an appropriate level of expertise to confirm that it is of an acceptable scientific standard, however I have significant reservations, as outlined above.

# Reviewer report: Reviewer response for version 3

**DOI:** 10.21956/mep.20680.r35641

**Published:** 2024-01-30

**Authors:** Elizabeth Brondolo

**Affiliations:** 1 St. John's University, New York, USA

The aim of this interesting essay is to describe and promote interventions that can improve end of life communication between physicians and patients/families. The essay is clear and many of the points are well formulated. But there are several difficulties which undermine the effectiveness of the essay.

 First, the author is trying to make too many points: 1) the value of understanding emotions that may lie beneath conscious awareness, 2) promotion of the Verbatim methodology, 3) discuss the notion of compulsory DoctorBodyCam or other interventions which require 3 ^rd^ party review of prognostic communication, and 4) evaluation of individual differences in provider capacity to recognize and communicate their own internal experiences as they influence others. Achieving any one of these aims would be commendable, but trying to include all four in one essay makes the essay unwieldly and less accessible.

 Second, the notion of increasing awareness of non-conscious emotions and motivations is introduced in a moving way in the essay. However, the author does not refer to any of the original theory, research and practice which supported the development of these ideas and the Verbatim approach. These are standard training tools in clinical psychology (since Freud). Writing the “the patient said, I said” ideas and considering them for interpretation of one’s own motives and ideas is a longstanding approach to training clinicians. These ideas have also been used in research on expressive writing by James Pennebaker and others. The notion of the non-conscious themes or schemas which structure our self-evaluation and perception of and relations with others have been well-articulated by Young (framed in the context of schema therapy) or Baldwin. The author links these ideas to anti-racism efforts, and the application of the notion of schemas to relationships affected by racial discrimination has been addressed by Brondolo. The paper would be improved and made both more scholarly and more accessible to a broader audience, if the author focused on highlighting the importance of examining unspoken emotions in the context of end-of-life care and placed these ideas in context of the larger literature.

 Third, as the author is describing personal experiences of others (i.e., his sister), an explicit statement that she has provided permission to share this information would be helpful. Although Dr. Gawande is a public figure and may be accustomed to being evaluated in a public forum, some moderating of the language used to describe his motivations may be helpful. Maybe the author could acknowledge the hypothetical nature of the speculations more clearly and tone down the tone a little.

 Fourth, physicians are under tremendous time pressure and often receive minimal support from others who are also under great time pressure. Expediency may partly drive their reactions to patients’ questions. Therefore, the requirement to include a neutral third party examiner is valuable. But the implementation of this idea will be so complex across institutions, even though the program is already being piloted. This idea deserves its own essay.

 Finally, there is a wealth of research on individual differences in the capacity to be introspective and empathic. It might be useful to consider this work as well.

 Overall, this paper makes many important points. But refining the aim, streamlining the approach, and embedding the ideas in the scholarship that supports them would improve the paper.

Is the case presented with sufficient detail to be useful for teaching or other practitioners?

Yes

Is the work clearly and accurately presented and does it cite the current literature?

No

If applicable, is the statistical analysis and its interpretation appropriate?

Not applicable

Is the case’s background and context in sufficient detail?

Yes

Are all the source data underlying the results available to ensure full reproducibility?

Yes

Are the conclusions drawn adequately supported by the results?

No

Reviewer Expertise:

behavioral medicine

I confirm that I have read this submission and believe that I have an appropriate level of expertise to confirm that it is of an acceptable scientific standard, however I have significant reservations, as outlined above.

## References

[1] : Racism and mental health: Examining the link between racism and depression from a social cognitive perspective. 2016; 10.1037/14852-006 109-132 10.1037/14852-006

[2] : Relational schemas and the processing of social information. *Psychological Bulletin* .1992;112(3) : 10.1037/0033-2909.112.3.461 461-484 10.1037/0033-2909.112.3.461

[3] : The Experience of Empathy in Everyday Life. *Psychol Sci* .2021;32(8) : 10.1177/0956797621995202 1198-1213 10.1177/0956797621995202 34241543 PMC13038167

[4] : Opening up: The healing power of expressing emotions (Rev. ed.). *Guilford Press* .1997; Reference source

[5] : Schema therapy: A practitioner's guide. *Guilford Press* .2003; Reference source

# Reviewer report: Reviewer response for version 3

**DOI:** 10.21956/mep.20680.r35194

**Published:** 2024-01-30

**Authors:** Sarah Shepherd

**Affiliations:** 1 The University of Manchester, Manchester, England, UK

I read this article with great interest, thank you for asking me to review it. I don’t think I have ever read an article like it, and so my comments are mainly around clarifying some of the aspects discussed, mainly because many of them are quite complex. I hope I can provide the perspective of an interested but less well versed reader.

 Overall I think the concept of the paper is excellent and I agree with every premise. I did find it complex to read and although this may be a stylist point there are areas throughout where I wondered if aspects could be simplified, or briefer.

 My comments:

- In abstract: Methods, not just granting physicians access, educators and HCPs might find it interesting so suggest changing ‘to grant access to’ with deletion of physicians.
- Introduction: clear overall concept, could be more concise but this is up to the author's style.
- Methodology: ‘…education module for emotion-centric **sle** of conversations’ – is this supposed to say ‘style’?
- Could you clarify your relationship with the ‘spiritual care team’ – are you a member? Are they aware of this publication?
- Could some explanation of what the verbatim module is be given before case studies are presented? A factual brief protocol would be helpful to set the scene.
- I read with fascination the case study and commend the raw honest account described.
- The hypothetical aspects were still interesting, less engrossing than your lived experience but made a point about how the verbatim exercise could be used.
- I wasn’t clear how the doctor body cams could prevent miscommunication – how would they be used? Are they literally cameras doctors wear?
- I’m not clear how healthcare notaries work? Are they present for the interaction?

Is the case presented with sufficient detail to be useful for teaching or other practitioners?

Yes

Is the work clearly and accurately presented and does it cite the current literature?

Yes

If applicable, is the statistical analysis and its interpretation appropriate?

Not applicable

Is the case’s background and context in sufficient detail?

Yes

Are all the source data underlying the results available to ensure full reproducibility?

Yes

Are the conclusions drawn adequately supported by the results?

Yes

Reviewer Expertise:

clinical communication education, psycho-oncology, shared decision making

I confirm that I have read this submission and believe that I have an appropriate level of expertise to confirm that it is of an acceptable scientific standard, however I have significant reservations, as outlined above.

# Reviewer report: Reviewer response for version 3

**DOI:** 10.21956/mep.20680.r33660

**Published:** 2024-01-30

**Authors:** Celina Carter

**Affiliations:** 1 SE Research Centre, Toronto, Ontario, Canada

Thank you for the opportunity to review this paper on an important topic - the need to improve the way clinicians discuss EOL with dying patients and their family/friends. The vulnerability and learnings shared are important and relevant for many practicing in healthcare. The need for emotional awareness of reactions has a long tradition in many disciplines and continues to be important to the field of EOL care.

 Please see my detailed comments to improve the rigor and quality of this autoethnography.

- This work is described as an autoethnography yet the word "we" is used. Please make clear who is speaking when and how this is consistent with the methodology discussed. Consistently write in the first person. Do not refer to "the author" in a subjective autoethnography.
- There is a need to tone down claims through the article as they read as untrue. For example, "modern medicine has no reliable way of getting to the bottom of such unthinkable communication failures". Part of modern medicine is research, which includes social theory and ethnographic study. Another example, "Healthcare’s only widespread communication training" yet there is lots of communication training all over the world. Be context specific - widespread where?
- The introduction needs to be more strongly rooted in the literature to frame the problem. Consider reviewing articles such as Granek *et al.* (2012 ^1^).
- The article needs to be reframed as one clinician's experience in training, their opinions, and what it offered their practice rather than making broad claims about training in EOL conversation and reflexivity globally. Self-reflective practice is a training in many disciplines not just spiritual care.
- "body cams" can be associated with policing technology, which is associated with violence towards many populations. Consider another metaphor (e.g., reflection in the mirror) or provide rationale for why this metaphor works.
- Many claims are opinions rather than fact. Reword to reflect this or provide citations for each claim such as "Medicine tends to leave the soft skills of conversational refinement to the chaplains and let the physicians just focus on the science". What about nurses, social workers, occupational therapists, etc.?
- Provide context and citation for "training for budding physicians are few and far between"
- Provide stronger rationale for using the methodology employed - why not an intervention study?
- Autoethnography is not consistent with objectivity.
- The description of autoethnography as subjective and interpretive should be applied to the writing throughout to increase rigor and coherence.
- "apply that insight to the analysis of other phenomena" - not to other phenomena but to elucidate a phenomena through subjective experience.
- The methodology is confusing. An autoethnography does not require a "case". No citation is provide for why case studies were introduced.
- The data source is unclear. What does this mean: "My case was narrated from previous “Verbatim” documents that I completed in the context of CPE Supervisory Training".
- To be consisent with autoethnography, the author should describe their experience and then bring this into conversation with Dr. Gawande's experience in the discussion - do not confuse two different methodologies by bringing in case studies.
- Discussion should start with a high level summary of key findings and clearly introduce literature for example, Loh's *et al.*'s work lacks detail and clear connection with your own experience of training.
- The discussion reads as an opinion piece that is not rooted in data, coherent with the methodology, or clearly connect to the broader literature. There is a need to thoughtfully link the author's emotional experience and learnings during training to research literature on EOL conversation training, curriculum, and perhaps other concepts that could be of interest such as transference, emotional intelligence, guilt, shame, etc. More context and understanding of the limitations of transferability of findings are also needed.
- There are problems that arise with mandating and legalizing - please discuss ideas that reflect the complexity and nuance of this practice.

Is the case presented with sufficient detail to be useful for teaching or other practitioners?

No

Is the work clearly and accurately presented and does it cite the current literature?

Partly

If applicable, is the statistical analysis and its interpretation appropriate?

Not applicable

Is the case’s background and context in sufficient detail?

No

Are all the source data underlying the results available to ensure full reproducibility?

No

Are the conclusions drawn adequately supported by the results?

No

Reviewer Expertise:

Qualitative research methods; EOL communication

I confirm that I have read this submission and believe that I have an appropriate level of expertise to state that I do not consider it to be of an acceptable scientific standard, for reasons outlined above.

## References

[1] : Nature and Impact of Grief Over Patient Loss on Oncologists' Personal and Professional Lives. *Archives of Internal Medicine* .2012;172(12) : 10.1001/archinternmed.2012.1426 10.1001/archinternmed.2012.1426 22732754

# Reviewer report: Reviewer response for version 3

**DOI:** 10.21956/mep.20680.r33659

**Published:** 2024-01-30

**Authors:** Poonam Goswami

**Affiliations:** 1 Texas Woman's University, Denton, Texas, USA

This article explains the hidden emotions of physicians when discussing end-of-life prognosis conversations.

 The author - a physician had explained with case studies and their own experience the emotions that were hindering them, and at the same time, used the ethnography to evaluate the Verbatim education module that can prepare physicians for underlying emotions during difficult prognosis disclosure.

 This self-study might encourage others to use the module and prepare them for difficult conversations, identifying their own emotions.

Is the case presented with sufficient detail to be useful for teaching or other practitioners?

Yes

Is the work clearly and accurately presented and does it cite the current literature?

Yes

If applicable, is the statistical analysis and its interpretation appropriate?

Not applicable

Is the case’s background and context in sufficient detail?

Yes

Are all the source data underlying the results available to ensure full reproducibility?

Yes

Are the conclusions drawn adequately supported by the results?

Yes

Reviewer Expertise:

End of Life

I confirm that I have read this submission and believe that I have an appropriate level of expertise to confirm that it is of an acceptable scientific standard.

# Reviewer report: Reviewer response for version 3

**DOI:** 10.21956/mep.20680.r32508

**Published:** 2022-08-25

**Authors:** Joseph T. S. Low

**Affiliations:** 1 Marie Curie Palliative Care Research Department, Division of Psychiatry, University College London, London, UK

I am happy with Dr Stonestreet's response to my last comments. Apologies for the slight confusion in my last review. Dr Stonestreet had addressed my initial queries, but in re-reading the manuscript, there were further queries I identified. However, at this point, these have been addressed and I have no further comments. I wish him all the best in his future work.

Is the case presented with sufficient detail to be useful for teaching or other practitioners?

Yes

Is the work clearly and accurately presented and does it cite the current literature?

Yes

If applicable, is the statistical analysis and its interpretation appropriate?

Not applicable

Is the case’s background and context in sufficient detail?

Yes

Are all the source data underlying the results available to ensure full reproducibility?

Yes

Are the conclusions drawn adequately supported by the results?

Yes

Reviewer Expertise:

Delivery of psychological care in palliative care population;  delivery of supportive and palliative care  in non-malignant disease; use of mixed methodologies; qualitative methodologies.

I confirm that I have read this submission and believe that I have an appropriate level of expertise to confirm that it is of an acceptable scientific standard.

# Reviewer report: Reviewer response for version 2

**DOI:** 10.21956/mep.20585.r32320

**Published:** 2022-07-22

**Authors:** Joseph T. S. Low

**Affiliations:** 1 Marie Curie Palliative Care Research Department, Division of Psychiatry, University College London, London, UK

Dear Editor, 

 Thank you for your detailed response from Dr Stonestreet following my original comments. I have read these and have re-read the revised manuscript. I am happy that the author has addressed my queries and I am happy to recommend this for indexing. 

 However, in re-reading the  manuscript, there is one point I need some clarification on. I was interested in the idea of the third-party communication support (DoctorBodyCam.com), but when I tried to search the website, I couldn't easily access it. Can I ask if the organisation who run this programme are a 'not for profit' organisation? Are there any financial incentives gained in recommending this specific intervention? 

 Apologies for not spotting this earlier. 

Is the case presented with sufficient detail to be useful for teaching or other practitioners?

Yes

Is the work clearly and accurately presented and does it cite the current literature?

Yes

If applicable, is the statistical analysis and its interpretation appropriate?

Not applicable

Is the case’s background and context in sufficient detail?

Yes

Are all the source data underlying the results available to ensure full reproducibility?

Yes

Are the conclusions drawn adequately supported by the results?

Yes

Reviewer Expertise:

Delivery of psychological care in palliative care population;  delivery of supportive and palliative care  in non-malignant disease; use of mixed methodologies; qualitative methodologies.

I confirm that I have read this submission and believe that I have an appropriate level of expertise to confirm that it is of an acceptable scientific standard, however I have significant reservations, as outlined above.

# Response

**Published:** 2022-08-04

**Authors:** John Stonestreet

**Affiliations:** Hopecare, USA

Dear Dr Low,

 My apologies in advance of this response that we are communicating across countries with different spellings for words you have used such as organisation, programme, etc.

 Thank you so much for your approval of how I addressed your queries in version 2 of my article.

 Thank you also for your stated interest in my idea of third-party communication support that I referenced in the Abstract as being accessible via DoctorBodyCam.com and that I cited throughout the paper as citation #34. Regarding your questions concerning this reference, the DoctorBodyCam.com domain name never pointed to any organizational website or existing program. It was simply forwarded to my peer reviewed article in the Journal of Health Ethics introducing a vision for the “Doctor Body Cam” protocol which again is cited throughout this article as citation #34 and is accessible online here: https://aquila.usm.edu/ojhe/vol17/iss1/7/. The reason you were not able to access the intuitive link is because I am in the process of renegotiating the renewal of multiple domain names that forward to different journal articles I have written, such as, for example, the domain “WhyDidHeSayThat.com” which pointed to this article that you are reviewing, as well as my domain “EOLdisparities.com” which pointed to my article: “Honoring Black Hopes: How to Respond When the Family is Hoping for a Miracle,” which is available online here: https://f1000research.com/articles/11-268. In due time, I expect for all of these domains to be renewed and point to the peer reviewed articles to which they provide intuitive access. However, since they are not currently active, I have made the following changes to my new version (version 3) of this article: First, I deleted the “DoctorBodyCam.com” url that you referenced in the Abstract and replaced it with a direct link to the referenced article. Second, I also deleted my mention of the “WhyDidHeSayThat.com” url at the beginning of the Abstract.

 Regarding your question of whether the organization that runs the "Doctor Body Cam" program is a nonprofit, there are multiple clarifications embedded here that I wish to address: First, while I did properly disclose with this article the competing interest (which you will see just below the Abstract) that I have as founder of Hopecare, I should clarify that when you refer to this “program” and “financial incentives,” these are future possibilities, not present realities. As a visionary idea person, I publish regarding ideas that I believe should come to concrete realization in healthcare. And as an innovator by nature, I have hopes that my work will find esteemed collaborators such as yourself and eventually come to fruition as concrete realities, and Hopecare exists as a vehicle through which some of these things may eventually materialize, God willing. But to be clear, Hopecare currently has zero revenue, and while it has not yet received its nonprofit designation, it will in due time.

 Returning to your stated interest in the “Doctor Body Cam” protocol, I invite you to read the article in my citation #34, found here: https://aquila.usm.edu/ojhe/vol17/iss1/7/. And I would absolutely love to hear your feedback on that vision, whether through this reviewing medium or via direct communication.

 Finally, I wish to clarify a potential discrepancy in your official response to this article as a peer reviewer. I know that this is a newish model for open, public peer review, and I want to add one clarification on how it works. I noticed that you stated the following in your latest review: 

 “I am happy that the author has addressed my queries and I am happy to recommend this for indexing.”

 To be clear, for this article to pass peer review, it would be helpful if you could update your official review status from the “question mark” icon (which represents approval with significant reservations) to the “check mark” icon (which represents approval without reservations or with smaller reservations).

 With gratitude for your kind consideration, 

 John Stonestreet, PhD

# Reviewer report: Reviewer response for version 1

**DOI:** 10.21956/mep.20456.r32094

**Published:** 2022-06-23

**Authors:** Joseph T. S. Low

**Affiliations:** 1 Marie Curie Palliative Care Research Department, Division of Psychiatry, University College London, London, UK

This manuscript aims to illustrate how the use of Spiritual Care 'Clinical Pastoral Education' (CPE) "Verbatim" practice can support clinicians to have difficult conversations about prognosis and end of life discussion with their patients by enabling clinicians to explore how their own emotions can hamper their own functioning in having these conversations.

 The author uses a methodology known as autoethnography. The author gives a good explanation of what autoethnology is and how it  is different from other social science methods. 

 I found the methodology section clear to follow, as was his selection of cases.

 In presenting his findings, I am happy with how the author relates his experiences with his clients to his CPE Verbatim training. The author also utilises two contrasting cases (one involving an unnamed ICU doctor reacting sarcastically to a miracle hoping family; the other Dr Gawande end-of life prognosis talk, and attempts to illustrate how CPE Vebatim training could have supported  clinicans in reconstructing their conversation with these patients. The author concludes that CPE Verbatim module could be a useful addition to the medical curriculum, but also acknowledges that its impact will vary depending on the 'physician in training's willingness to engage in the process'. 

 Overall, the manuscript was well-written, generally easy to follow, with lots of insights and reflections which I am sure many clinicians will be able to relate to. However, there are certain points that I was not clear about: 

1. In presenting the section 'Case studies', it is not clear how the different sub-sections (my case "unearthing hidden shame", "outward-directed emotions: The unnamed ICU Doctor case", 'inward emotions and how to identify them", etc., etc.)  arose. I think readers would appreciate an introductory sentence that you intend to present your cases in this way. Alternatively, I note that you mention this in your section "selection of case studies". I suggest that you are more transparent in your signposting, by making specific reference to your sub-sections in the section "selection of case studies'. 
2. In your introduction, you talk about 'conversation analysis'. Can you either rephrase this to analysing conversations or alternatively define what you mean by 'conversation analysis'? Some readers may be confused in thinking that you used a qualitative methodology known as 'conversation analysis' - an approach to the study of social interaction, embracing both verbal and non-verbal conduct, in situations of everyday life and initially proposed by Harvey Sacks, Emanuel Schegloff and Gail Jefferson. 
3. You mentioned some of the implications for medical education, but I would liked some reflections on the limitations of your analysis and interpretations, and what should research be looking at.    .

Is the case presented with sufficient detail to be useful for teaching or other practitioners?

Yes

Is the work clearly and accurately presented and does it cite the current literature?

Yes

If applicable, is the statistical analysis and its interpretation appropriate?

Not applicable

Is the case’s background and context in sufficient detail?

Yes

Are all the source data underlying the results available to ensure full reproducibility?

Yes

Are the conclusions drawn adequately supported by the results?

Yes

Reviewer Expertise:

Delivery of psychological care in palliative care population;  delivery of supportive and palliative care  in non-malignant disease; use of mixed methodologies; qualitative methodologies.

I confirm that I have read this submission and believe that I have an appropriate level of expertise to confirm that it is of an acceptable scientific standard, however I have significant reservations, as outlined above.

# Response

**Published:** 2022-06-28

**Authors:** John Stonestreet

**Affiliations:** Hopecare, USA

Dear Dr. Low, Thank you for taking the time and the care to offer your thoughtful review of my paper. I am very grateful to you for lending your expertise and providing invaluable guidance by offering three helpful suggestions for the betterment of this article. Out of respect for your valuable time, I have numbered and detailed my three responses here so that you can gain a full grasp of my edits without having to reread or even return to consult the updated article. I hope you will see my edits favorably and be in a position to update your review designation by offering your approval of this article without reservations. Sincerely,  John Stonestreet  PS: Please accept my appologies if the software tool I am pasting this content into jumbles some of the formatting which will not be jumbled in the article itself.

1. You pointed out that in my presentation of the “Case studies”, it is not clear how the different sub-sections arose. So you suggested that I be more transparent in my signposting, by making specific reference to the subsections in my “selection of case studies” discussion. 

To illustrate how I worked this suggestion into that section, I am providing two sentences from the revised text here. The first sentence below is my original introductory sentence for the “selection of case studies” section. The second sentence below is the one I have added to fulfill the requirement of “making specific reference to subsections in my ‘selection of cases’ discussion”. Here are the two sentences: "I selected my own case study not only because it illustrates the inner workings of CPE “Verbatim” practice, but also because, as you will see, some of the emotional dynamics revealed in my case are relevant to my analysis of Dr. Atul Gawande’s case. With that in mind, I take the liberty of using some descriptive subsections in my presentation of the cases to readers in order to highlight some conceptual issues, most especially those that ultimately culminate in the application of my case analysis to Gawande’s case analysis, namely the 'inner' emotion of 'hidden shame'."

1. You pointed out that my reference to “conversation analysis” in the introduction could be confused with the qualitative methodology that goes by the same name, so it was suggested that I rephrase this to “analyzing conversations.” In response to this suggestion, I did a search on “conversation analysis” and found that, including the Abstract, there were 9 instances of this nomenclature in the article, all of which I have rephrased. 
2. You pointed out that in addition to my mentions of the implications for medical education, it would be helpful if I also reflect on any limitations of my analysis and interpretations, and also what future research should be looking at. 

At the very end of the article, I added the section: “Limitations and Future Research,” which I have pasted below: 

 "Limitations and Future Research Now, I will address the limitations of my analysis/interpretations and corresponding suggestions for future research. Perhaps the most obvious limitation of what I have put forth here is that it is largely hypothetical in nature. I would like to contend that, however hypothetical it may be, not only is my analysis accurate, but it can even function as an interpretive key that unlocks the mysteries behind the two most common and impactful emotionally-driven communication failures affecting the quality and cost of end-of-life healthcare. And if that is not enough, I also claim to have developed novel interventions that solve both of these paradigmatic problems. ^22,34^ Given such a wide gap between the hypothetical nature of my analysis and my bold claims for its significance, the need for implementation research is clear.  But what kinds of implementation research might I suggest? Notice that in my bold claim for the significance of this article, I only referred to it as an “interpretive key.” Notice also that my "novel interventions" citations do not include a citation for "Verbatim" education modules. Indeed the primary intervention I would suggest for a randomized clinical trial (RCT) in response to the hidden shame blocking physicians’ terminal prognosis disclosures (that was revealed in this article) is not the “Verbatim” education exercise detailed here but the third-party “Doctor Body Cam” protocol ^34^ referenced above.  While I would love nothing more than to engage in the sacred task of interacting personally with medical students/residents/fellows who are open to testing out the “Verbatim” module and reporting back on their experience (which itself is an important form of research), I do not see this exercise as the best approach to large scale solvency for the emotionally-driven physician communication failures discussed in this article. This is due to the second limitation to my analysis in this article which is closely tied to my point three above that “there will be a variation in the extent to which each physician is open to engaging [the ‘Verbatim’ exercise] to maximum benefit.”  While it may seem like a subtle distinction, I want to take a step further and add that in addition to physicians’ openness to engaging the “Verbatim” exercise to maximum benefit, there is also the more fundamental limitation of physicians’ capacity for doing so. Let me explain. In my hypothetical analysis in the cases above, I assume an “aha” moment where the doctor “gets it,” and perhaps even more importantly, I also imply an assumption that this will translate into concrete changes in clinical practice, where the physician sees the detrimental emotion arising in real-time, side-steps and brackets that reflex, and then successfully self-initiates an effective conversational reroute. Could Dr. Gawande make that leap? Absolutely. His body of work clearly indicates that he already has. And the same goes for my reference to Dr. Zitter who confessed to the hero complex that previously drove her irrational interventions of harmful heroics. ^27 ^But Dr. Gawande and Dr. Zitter are not your average physicians. They are thought leaders and exceptional superstars to say the least. In pointing that out, I am not making a dig at the intelligence of the average doctor. More fundamentally, I am commenting on the reality of what doctors are trained to do: fight disease. Imagine for a moment that doctors are wind-up toy cars. When you wind them up and set them down, what do they do? They fight disease.  Both this observation and the related question of "capacity" I have borrowed from two forbearers. Twentieth century medical ethicist, Paul Ramsey, in his classic text, The Patient as Person, ^45^ identifies the “question of questions” for end-of-life medical decision making within the doctor-patient/family relationship. Loosely paraphrased, the question is this: Can we reasonably expect to find within the same person, both the main vocation to fight disease and also the added vocation of taking the initiative with patients and families to introduce the question of when it is time to stop fighting disease and focus on putting affairs and relationships in order in preparation for the best possible experience of death? In addition to Ramsey’s “question of questions,” which I think raises the issue of physicians’ prognostic capacity, I borrow the provocative term, “capacity” itself, from another twentieth century medical ethicist, Dr. Jay Katz. In response to an early 1980s United States Presidential Commission calling for shared [medical] decision making (SDM) in the doctor-patient relationship, Dr. Katz names physicians’ “incapacity" for truth-telling due to most “doctors’ disregard of uncertainty.” ^46^ Here, it seems to me that Katz is provisionally answering Ramsey's "question of questions" in the negative. Trained and practicing as a psychiatrist, Katz would not throw around the term “incapacity” lightly. Also trained as a lawyer, Katz understood the legal implications of the medical designation of incapacity or "incompetence," a term he also uses in calling his fellow physicians' prognostic and SDM capabilities into question. Katz knows that if patients who are declared "incompetent" for the task of medical decision-making are replaced by third-party "surrogate" decision-makers, then the same might necessarily follow for physicians, the majority of whom have an incapacity for prognostic truth-telling (Regarding this prevalent incapacity, Dr. Nicholas Christakis wrote an entire book detailing his extensive research on the prevalence of physicians’ prognostic shortcomings ^47^). And Katz's legal chops would also make him inherently sensitive to the dangers of replacing physicians' role in sharing end-of-life decision-making with patients and families. Namely, there could be a legal danger of removing the task of medical decision-making from the doctor-patient/family relationship altogether and placing it into the hands of third-party bureaucracies who are external to the doctor-patient/family relationship. I think this is why Katz suggests that medicine resort to "limping" very slowly and everso cautiously in the direction of figuring out exactly how to comply with that 1980s presidential commission's vision for the future of medical decision-making.  Now nearly forty years later, it might be said that American medicine has not limped all that far, the growth of palliative care and hospice notwithstanding. This is why it again bears repeating that Dr. Al-Samkari finds his fellow physicians' prognostic disclosure capabilities still limping in the "dark ages." ^12^ When Al Samkari suggests that if doctors are not lying to their patients then they are lying to themselves, that assertion begs the question of capacity.  This is not to say that medical students, residents, and fellows have nothing at all to gain from "Verbatim" education modules, but that complete solvency to the problems raised in this article requires a multipronged approach. As stated above, the linchpin is the third-party “healthcare notary” who notarizes shared understanding between doctors, patients, and families for end-of-life and earlier difficult healthcare issues. ^34^ To summarize my vision for future research, I seek collaborators for the following three forms of research. First, I would like to deliver a Visiting Lecture on this article for medical students and Grand Rounds for practicing physicians. Afterwards, I would like to circulate a survey for feedback on the training method as well as the underlying conceptual issues. Secondly (and I envision that the first suggestion might most naturally lead to the second), I would like to pilot-test the Verbatim practice detailed above with patient-facing medical students, residents, and fellows, circulating a follow-up survey, especially soliciting open-ended responses (I am also open to collaborators' priorities for surveys). Third, I seek collaborators for a program of research that moves in the direction of major RCTs testing the quality and cost outcomes of third-party healthcare notaries inserted into the doctor-patient/family relationship for medical decision-making. My main role in the research collaboration would be to provide the training for the third-party healthcare notaries delivering the intervention.  Why am I confident that after nearly forty years of limping through the wilderness in search of the Promised Land of prognostic truth-telling and shared decision-making (SDM), that it is now time to take these ideas and run with them? Because the role of the “healthcare notary” ^34^ walks the tightrope that Dr. Katz suggested we limp toward mastering, without erring on either side of that fine line and falling into the abyss. Here's why: the healthcare notary addresses the issue of physicians' widespread emotional incapacity for prognostic truth-telling and SDM without overcorrecting for that problem by removing the locus of the medical decision outside of the doctor-patient/family relationship. This is because the notary is not imposing some external criteria for supposedly valid healthcare decisions but instead serving as a witness to the reality of a shared understanding of critical issues, between doctor, patient, and family. The healthcare notary's only role then is to act as a structural facilitator to the success of the all-important and irreplaceable doctor-patient/family relationship for prognosis and SDM. A specific example of what that might look like is found in the “Doctor Body Cam” protocol piece. ^34^ How does this vision fit into the current landscape for SDM research in the health services research community and the related and long awaited physician uptake of SDM more broadly? For readers not heavily embedded in the SDM research community it bears mentioning that some of its leaders published an article ^48^ in 2020 predicting a "dystopian future" for SDM if it continues along its current trajectory. That trajectory lacks a viable pathway to physicians' widespread adoption of SDM, making many if not all of the SDM research community's marvelously constructed "decision aids" significantly underutilized.  This is because, in my opinion, the SDM research community looks almost exactly like the "research ethics" community would look like in a world without the Institutional Review Board (IRB). How can you get anyone to adopt your suggestions for ethical practice without a mandate? This is why the suggestions of that 1980s presidential commission on SDM have gone largely ignored for nearly 40 years: there was/is no mandate. For “research ethics,” the IRB was a mandate because you cannot conduct research without it. Requiring *notarized* prognosis disclosure and SDM before being legally permitted to initiate “informed consent” would be a mandate. Then suddenly all of the marvelous, context-specific "decision aids" created by the SDM research community would be grabbed up and used everywhere. And that for the same reason that researchers do whatever is necessary for IRB approval: it is mandated as a requirement for moving forward.  Here's to a utopian future for SDM with prognostic truth-telling as its first step."
